# Natural Product-Based Pesticide Discovery: Design, Synthesis and Bioactivity Studies of *N*-Amino-Maleimide Derivatives

**DOI:** 10.3390/molecules23071521

**Published:** 2018-06-24

**Authors:** Xiangmin Song, Chunjuan Liu, Peiqi Chen, Hao Zhang, Ranfeng Sun

**Affiliations:** 1 Institute of Tropical Agriculture and Forestry, Hainan University, Haikou 570228, China; 18898958572@163.com; 2 School of Chemical & Chemical Engineering, Inner Mongolia University, Hohhot 010021, China; 17330926558@163.com (C.L.); chenpq17@lzu.edu.cn (P.C.); haozhang@imu.edu.cn (H.Z.)

**Keywords:** maleimide, linderone, methyllinderone, antifungal activity

## Abstract

Natural products are an important source of pesticide discovery. A series of *N*-amino-maleimide derivatives containing hydrazone group were designed and synthesized based on the structure of linderone and methyllinderone which were isolated from *Lindera erythrocarpa* Makino. According to the bioassay results, compounds **2** and **3** showed 60% inhibition against mosquito (*Culex pipiens pallens*) at 0.25 µg·mL^−1^. Furthermore, the results of antifungal tests indicated that most compounds exhibited much better antifungal activities against fourteen phytopathogenic fungi than linderone and methyllinderone and some compounds exhibited better antifungal activities than commercial fungicides (carbendazim and chlorothalonil) at 50 µg·mL^−1^. In particular, compound **12** exhibited broad-spectrum fungicidal activity (>50% inhibitory activities against 11 phytopathogenic fungi) and compounds **12** and **14** displayed 60.6% and 47.9% inhibitory activity against *Rhizoctonia cerealis* at 12.5 µg·mL^−1^ respectively. Furthermore, compound **17** was synthesized, which lacks N-substituent at maleimide and its poor antifungal activity against *Sclerotinia sclerotiorum* and *Rhizoctonia cerealis* at 50 µg·mL^−1^ showed that the backbone structure of *N*-amino-maleimide derivatives containing hydrazone group was important to the antifungal activity.

## 1. Introduction

**Figure 1:**
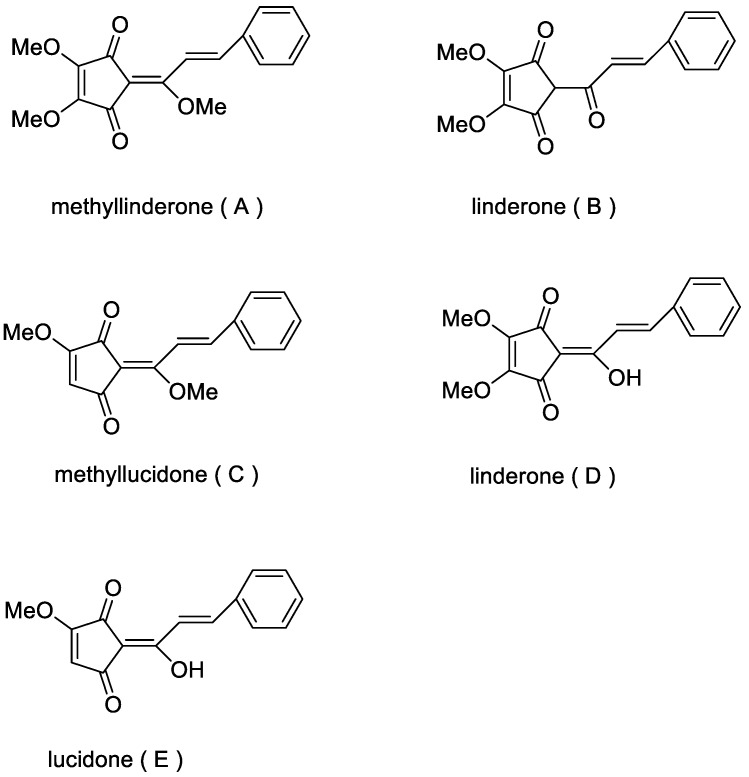
Chemical structures of methyllinderone (**A**), linderone (**B**), methyllucidone (**C**), linderone (**D**), lucidone (**E**).

Chitin is a unique component of the fungal cell wall and shells of crustaceans, but it is absent in vertebrates, mammals, and humans [1]. Chitin synthase is thus an attractive molecular target for developing fungicides and insecticides. Natural products are an important source for drug and pesticide discovery. *Lindera* species (Lauraceae) have rich chemical compositions and pharmacological activities [2]. Methyllinderone (**A**) and linderone (**B**), which were classic inhibitors of chitin synthetase (Figure 1), were isolated from *Lindera erythocarpa* Makino (Lauraceae) and exhibited inhibitory activity against chitin synthase 2 (CaCHS2p) with IC_50_ value of 23.3, 21.4 µg·mL^−1^ respectively, which was better than polyoxin D (70.0 µg·mL^−1^) and nikkomycin Z (176.0 µg·mL^−1^) [3]. In 2015, Seok-Hee Lee et al. reported that methyllinderone (**A**) and methyllucidone (**C**) (Figure 1) exhibited juvenile hormone antagonistic activity against *Aedes aegypti* [4]. Sheng-Yang Wang et al. reported that linderone (**D**), methyllinderone (**A**), lucidone (**E**) and methyllucidone (**C**) (Figure 1) displayed good anti-inflammatory activity [5].

**Figure 2:**
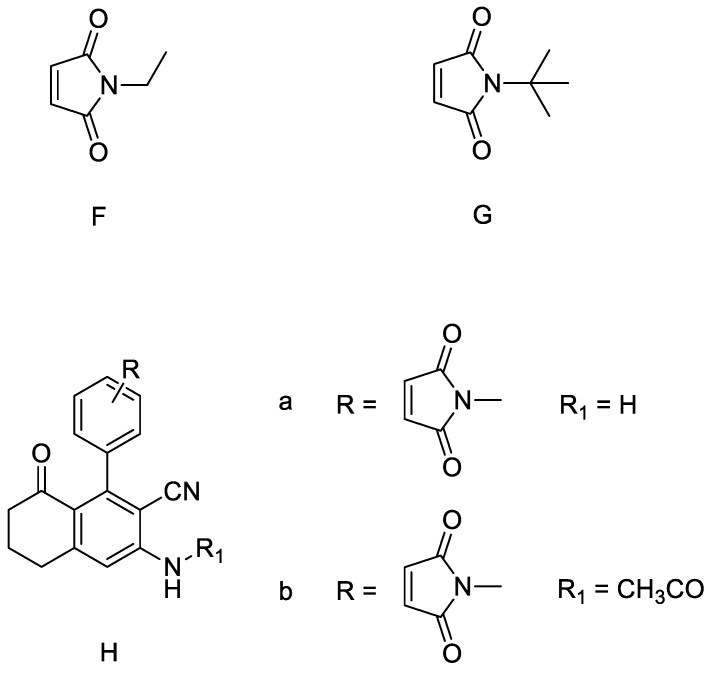
Structures of compounds with maleic amide fragment.

Interestingly, it was found that compounds containing a maleimide fragment also have chitin synthase and β-1,3-glucan synthase inhibitory activity according to the literature. For example, compounds **F** and **G** (Figure 2) inhibited β-1,3-glucan synthase with IC_50_ value of 8.5, 17.3 µg·mL^−1^, respectively [6]. In 2007, Atul R. Gholap et al. reported compound **H** (Figure 2) containing maleimide group performed over 91% inhibitory against chitin synthase, which exhibited comparable activity with nikkomycin Z (90%) [7].

**Figure 3:**
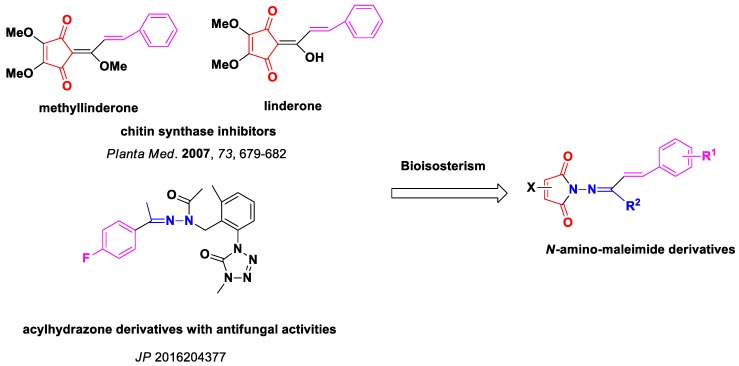
Design strategy for *N*-amino-maleimide derivatives.

Therefore, we focused on introducing maleimide fragments into natural products to obtain more efficient fungicides and insecticides. A series of *N*-amino-maleimide derivatives containing hydrazone group were designed based on the structure of methyllinderone, linderone and acylhydrazone derivatives with excellent antifungal activity (Figure 3) [8]. Then target compounds **1**–**16** were synthesized and their biological activities were evaluated.

## 2. Results

### 2.1. Synthesis

Different aryl-substituted unsaturated ketones, which were synthesized according to the literature [9], and *N*-amino-maleimide, which were produced in situ without purification [10], are refluxed in dry EtOH with a catalytic amount of *p*-toluenesulfonic acid to produce a variety of *N*-amino-maleimide derivatives containing hydrazone group (compounds **1**–**16**, Scheme 1) and NMR spectral information was listed (Figures S1–S34). Although the reaction produced good yields, the maleic anhydride and hydrazine hydrate cyclized to form a six-membered ring isomer (Figure 4) which is in accordance with previous report [10].

**Scheme 2:**
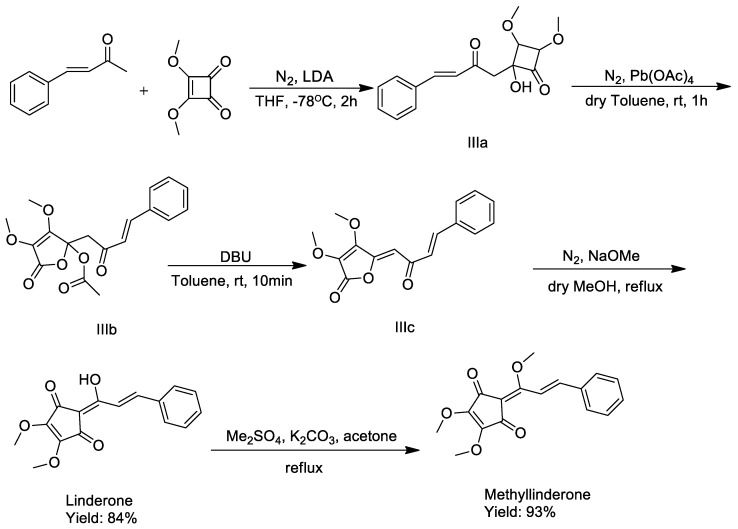
Synthetic route for linderone and methyllinderone.

At the same time, linderone and methyllinderone were synthesized according to the literature (Scheme 2) [11].

**Scheme 3:**
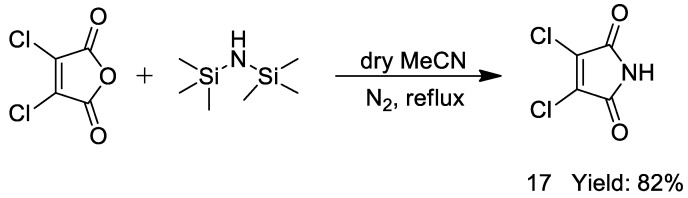
Synthetic route for compound **17**.

Finally, 3,4-dichloro-1*H*-pyrrole-2,5-dione (**17**) was synthesized to further illustrate the active functional group of compounds **1**–**16** (Scheme 3) [10].

### 2.2. Bioassays

#### 2.2.1. Stomach Toxicity against Oriental Armyworm (*Mythimna separata*)

It can be seen from Table 1 that some compounds have considerable insecticidal activities against oriental armyworm and seemed to display some electronic effects. For example, comparing the insecticidal activities of compounds **1**, **6**, **10**, we can easily find that when the phenyl ring (R = H) is unsubstituted, the electron-rich substituents of maleimide unit will significantly enhance the insecticidal activity of the compound, the insecticidal activity values of compounds **1** (X = H), **6** (X = 2-CH_3_), **10** (X = 2,3-Cl) are 25%, 65% and 20% respectively. When comparing two sets of the compounds substituted in maleinimide fragment **1**–**5** (X = H) or **6**–**8** (X = 2-CH_3_), it was found that they displayed different structure–activity relationships (SAR_S_). For example, when the maleimide units are unsubstituted (X = H), compound **2** (R = 3,5-CF_3_) and **3** (R = 2,4-Cl) displayed superior insecticidal activities (60%, 50% respectively) than compound **4** (R = 4-NO_2_) and **5** (R = 2-F) (10%, 25% respectively), whereas compound **6** (R = H) exhibited the best insecticidal activities when the substituent of maleimide units is methyl group (X = 2-CH_3_). In general, the insecticidal activity of the meta-substitute on the phenyl ring is superior to that of the ortho and para-substitute.

#### 2.2.2. Toxicity against Mosquito (*Culex pipiens pallens*)

Table 1 showed the larvacidal activities of the compounds **1**–**8**, **10** against mosquito. The bioassay results indicated that some compounds (compounds **2**, **3** and **8**) exhibited excellent larvicidal activities against mosquito, especially compounds **2** (X = H, R = 3,5-CF_3_) and **3** (X = H, R = 2,4-Cl) which showed 60% mortality even at 0.25 µg·mL^−1^. This seems to indicate that the substitution of the meta position on the phenyl ring or the substitution of the ortho and para positions will have a greater effect on the activity of the compounds.

#### 2.2.3. In Vitro Antifungal Activity

The fungicidal results of compounds **1**–**17** are listed in Table 2 and Table 3. Most compounds exhibited much better antifungal activities against fourteen phytogenic fungi than linderone and methyllinderone and some compounds exhibited better antifungal activities than commercial fungicides (carbendazim and chlorothalonil). All the compounds except compound **1**, **4**–**6** and **9** showed more than 50% inhibition rate against *Rhizoctonia cerealis* at 50 μg·mL^−1^ and six compounds (**10**–**13**, **14**–**16**) showed better antifungal activities than linderone (58.2%) and methyllinderone (74.5%), in particular compounds **12**, **14**, and **15** with 98.2%, 100.0%, 90.9% of inhibition rate respectively. When the concentration was adjusted to 25 μg·mL^−1^ and 12.5 μg·mL^−1^, the inhibition rate of compound **12** against *Rhizoctonia cerealis* was 95.8%, 60.6% and the inhibition rate of compound **14** against *Rhizoctonia cerealis* was 77.5%, 47.9% respectively (Table 3). Compound **2**, **4**, **6**, **7**, **10**, **14** and **16** showed more than 50% inhibition rate against *Botryospuaeria berengeriana* at 50 μg·mL^−1^, and five of them (compound **2**, **4**, **6**, **10** and **14**) were superior to linderone (57.1%) and methyllinderone (8.2%), for example, compound **4** exhibited 89.3% inhibitory activity. Compound **10**–**12** and **14**–**16** showed more than 60% inhibition rate against *Sclerotinia sclerotiorum* at 50 μg·mL^−1^, which were even superior to chlorothalonil (<50%). Compound **8**, **11**, **12**, **15** and **16** showed more than 50% inhibition rate against *Cercospora arachidicola* at 50 μg·mL^−1^, which were even superior to carbendazim (<50%). Compound **4**, **12**, **15** and **16** displayed more than 50% inhibition rate against *Helminthosporium maydis* at 50 μg·mL^−1^, which were superior to linderone (27.8%) and methyllinderone (38.9%). Compound **10**–**12** and **15**–**16** showed more than 50% inhibition rate against *Botrytis cinerea* at 50 μg·mL^−1^, which were superior to linderone (27.8%) and methyllinderone (38.9%). Compound **2**, **7**, **10**, **12** and **14**–**16** showed more than 50% inhibition rate against *Rhizoctonia solani* at 50 μg·mL^−1^, which were superior to linderone (28.4%) and methyllinderone (24.7%). Compound **12** and **14**–**16** showed more than 50% inhibition rate against *Phytophthora capsica* at 50 μg·mL^−1^, which were superior to linderone (27.8%) and methyllinderone (27.8%). In particular, compound **14** exhibited broad-spectrum fungicidal activities against test phytogenic fungi and even displayed much better antifungal activities against five fungi (*Botrytis cinereal*, *Fusarium oxysporum* f.sp. *cucumerinum*, *Cercospora arachidicola*, *Fusarium moniliforme* and *Phytophthora capsica*) than carbendazim (<50%). At the same time, we found that the substituents of maleimide have an important effect on the antifungal activity, for example, compound **12** (X = 2,3-Cl, R = 2,4-Cl) exhibits more power and broad-spectrum antifungal activity than compounds **3** (X = H, R = 2,4-Cl), **8** (X = 2-CH_3_, R = 2,4-Cl). Finally, in order to prove whether the maleimide has antifungal activity as reported in the literature [12], we synthesized compound **17** and then tested their inhibitory activities against *Sclerotinia sclerotiorum* and *Rhizoctonia cerealis*, which was 21.4%, 23.6% respectively at 50 μg·mL^−1^ (Table 3). This result indicates that the antifungal activities of compounds **1**–**16** are dependent by the entire pesticide molecule rather than the maleimide fragment.

## 3. Materials and Methods

### 3.1. Instruments

NMR spectra were obtained at 500 MHz using a Bruker avance III 500 spectrometer (Bruker Daltonics, Bremen, Germany) in CDCl_3_ or DMSO-d_6_ solution with tetramethylsilane as the internal standard. HRMS data were obtained on an Ultraflex III MALDI-TOF/TOF (Bruker Daltonics). The melting points were determined on an X-4 binocular microscope melting point apparatus (Beijing Tech Instruments Co., Beijing, China) and are uncorrected.

### 3.2. General Synthesis

All anhydrous solvents were dried and purified by standard techniques. Chemical materials purchased from Aladdin (Shanghai, China) or TCI (Shanghai, China) for analytical grade.

### 3.3. General Procedure for the Synthesis of Compounds ***1***–***16***

Unsaturated aldehydes or ketones and *N*-amino-maleimide products were prepared according to the literature [9,10]. A mixture of unsaturated aldehydes or ketones (3.0 mmol), *N*-amino-maleimide products (2 mmol) and *p*-toluenesulfonic acid (0.2 mmol) in dry ethanol (5 mL) was heated under reflux for 12 h with stirring. The organic layer diluted with EtOAc (20 mL), washed twice with a saturated NaHCO_3_ solution (20 mL) and brine (20 mL), dried with anhydrous Na_2_SO_4_ and evaporated in vacuo. Finally, the residue was purified by silica gel column chromatography (EtOAc/petroleum ether) to afford compounds **1**–**16**.

*1-(((E)-4-phenylbut-3-en-2-ylidene)amino)-1H-pyrrole-2,5-dione* (**1**). Pale yellow solid, mp 159–161 °C, yield = 67%. ^1^H-NMR (CDCl_3_) δ 7.37–7.29 (m, 5H), 6.89 (d, *J* = 10.2 Hz, 1H), 6.80 (d, *J* = 10.2 Hz, 1H), 6.06 (s, 1H), 5.39 (s, 1H), 2.53 (s, 3H); ^13^C-NMR (CDCl_3_) δ 154.1, 154.0, 137.0, 136.3, 135.8, 134.2, 128.9, 127.2, 110.8, 77.4, 77.1, 76.8, 15.2; HRMS (ESI): *m*/*z* calcd for C_14_H_12_N_2_O_2_ [M + H]^+^: 241.0977, found 241.0972.

*1-(((E)-4-(3,5-bis(trifluoromethyl)phenyl)but-3-en-2-ylidene)amino)-1H-pyrrole-2,5-dione* (**2**). Pale yellow solid, mp 122–125 °C, yield = 57%. ^1^H-NMR (CDCl_3_) δ 7.84 (s, 1H), 7.78 (s, 2H), 6.94 (d, *J* = 10.2 Hz, 1H), 6.83 (d, *J* = 10.2 Hz, 1H), 6.17 (s, 1H), 5.36 (s, 1H), 2.57 (s, 3H); ^13^C-NMR (CDCl_3_) δ 154.3, 154.0, 138.8, 138.6, 136.9, 133.9, 127.8, 123.0, 108.4, 77.3, 77.1, 76.8, 65.7, 15.2; HRMS (ESI): *m*/*z* calcd for C_16_H_10_N_2_O_2_F_6_ [M + H]^+^: 377.0719, found 377.0699.

*1-(((E)-4-(2,4-dichlorophenyl)but-3-en-2-ylidene)amino)-1H-pyrrole-2,5-dione* (**3**). Pale yellow solid, mp 143–144 °C, yield = 61%. ^1^H-NMR (CDCl_3_) δ 7.40 (s, 1H), 7.22 (d, *J* = 8.4 Hz, 1H), 7.01 (d, *J* = 8.4 Hz, 1H), 6.95 (d, *J* = 10.2 Hz, 1H), 6.86 (d, *J* = 10.2 Hz, 1H), 6.38 (s, 1H), 5.39 (s, 1H), 2.48 (s, 3H); ^13^C-NMR (CDCl_3_) δ 153.9, 137.7, 136.7, 134.8, 133.7, 132.9, 131.8, 130.1, 127.9, 108.6, 77.3, 77.1, 76.8, 15.1; HRMS (ESI): *m*/*z* calcd for C_14_H_10_N_2_O_2_Cl_2_ [M + H]^+^: 308.0113, found 308.0096.

*1-(((E)-4-(4-nitrophenyl)but-3-en-2-ylidene)amino)-1H-pyrrole-2,5-dione* (**4**). Pale yellow solid, mp 172–174 °C, yield = 52%. ^1^H-NMR (CDCl_3_) δ 8.19 (d, *J* = 8.2 Hz, 2H), 7.48 (d, *J* = 8.2 Hz, 2H), 6.92 (d, *J* = 10.2 Hz, 1H), 6.82 (d, *J* = 10.2 Hz, 1H), 6.13 (s, 1H), 5.36 (s, 1H), 2.53 (s, 3H); ^13^C-NMR (CDCl_3_) δ 154.1, 153.9, 148.1, 142.9, 138.3, 136.8, 133.9, 128.2, 124.2, 109.0, 77.4, 77.2, 76.9, 15.2; HRMS (ESI): *m*/*z* calcd for C_14_H_11_N_3_O_4_ [M + H]^+^: 285.0750, found 285.0760.

*1-(((E)-4-(2-fluorophenyl)but-3-en-2-ylidene)amino)-1H-pyrrole-2,5-dione* (**5**). Pale yellow solid, mp 135–137 °C, yield = 63%. ^1^H-NMR (CDCl_3_) δ 7.31–7.26 (m, 1H), 7.10 (ddd, *J* = 29.9, 16.0, 7.0 Hz, 3H), 6.92 (d, *J* = 10.2 Hz, 1H), 6.84 (d, *J* = 10.2 Hz, 1H), 6.29 (s, 1H), 5.38 (s, 1H), 2.49 (s, 3H); ^13^C-NMR (CDCl_3_) δ 161.23, 159.3, 154.0, 137.4, 136.5, 133.8, 130.3, 127.7, 124.6, 116.2, 116.0, 109.3, 77.4, 77.1, 76.8, 15.1; HRMS (ESI): *m*/*z* calcd for C_14_H_11_N_2_O_4_F [M + H]^+^: 258.0799, found 258.0771.

*3-methyl-1-(((E)-4-phenylbut-3-en-2-ylidene)amino)-1H-pyrrole-2,5-dione* (**6**). Pale yellow solid, mp 143–144 °C, yield = 68%. ^1^H-NMR (CDCl_3_) δ 7.38–7.28 (m, 5H), 6.71 (d, *J* = 26.2 Hz, 1H), 6.04 (s, 1H), 5.35 (d, *J* = 21.6 Hz, 1H), 2.53 (d, *J* = 8.0 Hz, 3H), 2.13 (d, *J* = 30.7 Hz, 3H); ^13^C-NMR (CDCl_3_) δ 155.1, 154.4, 146.0, 144.4, 137.0, 136.2, 132.2, 130.4, 129.0, 128.6, 127.4, 127.1, 111.0, 110.6, 77.4, 77.1, 76.9, 16.9, 16.4, 15.4, 15.2; HRMS (ESI): *m*/*z* calcd for C_15_H_14_N_2_O_2_ [M + H]^+^: 277.0974, found 277.0969.

*1-(((E)-4-(3,5-bis(trifluoromethyl)phenyl)but-3-en-2-ylidene)amino)-3-methyl-1H-pyrrole-2,5-dione* (**7**). Pale yellow solid, mp 143–144 °C, yield = 68%. ^1^H-NMR (CDCl_3_) δ 7.83 (s, 1H), 7.77 (d, *J* = 5.8 Hz, 2H), 6.74 (d, *J* = 40.7 Hz, 1H), 6.15 (s, 1H), 5.33 (d, *J* = 21.8 Hz, 1H), 2.56 (d, *J* = 9.1 Hz, 3H), 2.16 (d, *J* = 37.4 Hz, 3H); ^13^C-NMR (CDCl_3_) δ 155.4, 155.0, 154.7, 154.3, 146.9, 144.3, 139.1, 138.7, 132.8, 132.4, 132.1, 130.0, 127.8, 124.2, 122.9, 122.0, 108.7, 107.9, 77.4, 76.9, 65.8, 65.4, 17.0, 16.4, 15.6, 15.4; HRMS (ESI): *m*/*z* calcd for C_17_H_12_N_2_O_2_F_6_ [M + H]^+^: 391.0875, found 391.0904.

*1-(((E)-4-(2,4-dichlorophenyl)but-3-en-2-ylidene)amino)-3-methyl-1H-pyrrole-2,5-dione* (**8**). Pale yellow solid, mp 143–144 °C, yield = 68%. ^1^H-NMR (CDCl_3_) δ 7.39 (t, *J* = 2.0 Hz, 1H), 7.21 (ddd, *J* = 8.3, 4.9, 2.1 Hz, 1H), 7.01 (dd, *J* = 12.4, 8.4 Hz, 1H), 6.76 (d, *J* = 25.1 Hz, 1H), 6.37 (d, *J* = 20.2 Hz, 1H), 5.35 (d, *J* = 37.7 Hz, 1H), 2.47 (d, *J* = 5.7 Hz, 3H), 2.16 (d, *J* = 32.3 Hz, 3H); ^13^C-NMR (CDCl_3_) δ 155.0, 154.3, 146.6, 144.1, 137.8, 134.8, 133.2, 132.9, 132.6, 132.2, 130.3, 129.9, 128.2, 127.9, 108.9, 108.1, 77.4, 77.1, 76.9, 17.0, 16.4, 15.3, 15.1; HRMS (ESI): *m*/*z* calcd for C_15_H_12_N_2_O_2_Cl_2_ [M + H]^+^: 323.0348, found 323.0355.

*4,5,6,7-tetrachloro-2-(((E)-4-phenylbut-3-en-2-ylidene)amino)isoindoline-1,3-dione* (**9**). Pale yellow solid, mp 212–213 °C, yield = 58%. ^1^H-NMR (CDCl_3_) δ 7.56 (d, *J* = 7.2 Hz, 2H), 7.47–7.34 (m, 3H), 7.31 (d, *J* = 16.5 Hz, 1H), 7.17 (d, *J* = 16.4 Hz, 1H), 2.20 (s, 3H); ^13^C-NMR (CDCl_3_) δ 176.1, 159.6, 140.5, 135.2, 130.0, 129.1, 127.6, 127.1, 126.9, 77.4, 77.1, 76.8, 16.4; HRMS (ESI): *m*/*z* calcd for C_18_H_10_N_2_O_2_Cl_4_ [M + H]^+^: 448.9388, found 448.9383.

*3,4-dichloro-1-(((E)-4-phenylbut-3-en-2-ylidene)amino)-1H-pyrrole-2,5-dione* (**10**). Pale yellow solid, mp 152–153 °C, yield = 62%. ^1^H-NMR (CDCl_3_) δ 7.39–7.31 (m, 5H), 6.10 (s, 1H), 5.49 (s, 1H), 2.56 (s, 3H); ^13^C-NMR (CDCl_3_) δ 149.5, 149.2, 139.4, 137.9, 137.3, 134.6, 129.3, 129.0, 127.7, 111.6, 77.4, 77.1, 76.9, 15.3; HRMS (ESI): *m*/*z* calcd for C_14_H_10_N_2_O_2_Cl_2_ [M + H]^+^: 308.0113, found 308.0093.

*1-(((E)-4-(3,5-bis(trifluoromethyl)phenyl)but-3-en-2-ylidene)amino)-3,4-dichloro-1H-pyrrole-2,5-dione* (**11**). Pale yellow solid, mp 152–153 °C, yield = 60%. ^1^H-NMR (CDCl_3_) δ 7.87 (s, 1H), 7.81 (s, 2H), 6.22 (s, 1H), 5.47 (s, 1H), 2.60 (s, 3H); ^13^C-NMR (CDCl_3_) δ 150.0, 149.2, 140.2, 139.2, 137.8, 137.5, 132.7, 132.4, 128.4, 123.5, 109.3, 77.4, 77.2, 76.9, 15.5; HRMS (ESI): *m*/*z* calcd for C_16_H_8_N_2_O_2_F_6_Cl_2_ [M + H]^+^: 466.9759, found 466.9751.

*3,4-dichloro-1-(((E)-4-(2,4-dichlorophenyl)but-3-en-2-ylidene)amino)-1H-pyrrole-2,5-dione* (**12**). Pale yellow solid, mp 189–191 °C, yield = 62%. ^1^H-NMR (CDCl_3_) δ 7.42 (d, *J* = 2.0 Hz, 1H), 7.27–7.25 (m, 1H), 7.05 (d, *J* = 8.4 Hz, 1H), 6.45 (s, 1H), 5.47 (s, 1H), 2.52 (s, 3H); ^13^C-NMR (CDCl_3_) δ 155.0, 154.3, 146.6, 144.1, 137.8, 134.8, 133.2, 132.9, 132.6, 132.2, 130.3, 129.9, 128.2, 127.9, 108.9, 108.1, 77.4, 77.1, 76.8, 16.4, 15.3, 15.1; HRMS (ESI): *m*/*z* calcd for C_14_H_8_N_2_O_2_Cl_4_ [M + H]^+^: 398.9232, found 398.9234.

*3,4-dichloro-1-(((E)-4-(4-nitrophenyl)but-3-en-2-ylidene)amino)-1H-pyrrole-2,5-dione* (**13**). Pale yellow solid, mp 161–163 °C, yield = 41%. ^1^H-NMR (CDCl_3_) δ 8.22 (s, 2H), 7.52 (d, *J* = 8.5 Hz, 2H), 6.20 (s, 1H), 5.48 (s, 1H), 2.57 (s, 3H); ^13^C-NMR (CDCl_3_) δ 154.1, 153.9, 148.1, 142.9, 138.3, 136.8, 133.9, 128.2, 124.2, 109.0, 77.4, 77.2, 76.9, 15.2; HRMS (ESI): *m*/*z* calcd for C_14_H_9_N_3_O_4_Cl_2_ [M + H]^+^: 352.9956, found 352.9970.

*3,4-dichloro-1-(((E)-4-(2-fluorophenyl)but-3-en-2-ylidene)amino)-1H-pyrrole-2,5-dione* (**14**). Pale yellow solid, mp 140–141 °C, yield = 64%. ^1^H-NMR (CDCl_3_) δ 7.33–7.29 (m, 1H), 7.18–7.13 (m, 2H), 7.07 (s, 1H), 6.33 (s, 1H), 5.47 (s, 1H), 2.53 (d, *J* = 1.5 Hz, 3H); ^13^C-NMR (CDCl_3_) δ 161.5, 159.5, 149.3, 139.7, 137.7, 130.8, 128.2, 124.8, 122.0, 116.4, 116.2, 110.2, 77.4, 77.1, 76.9, 15.3; HRMS (ESI): *m*/*z* calcd for C_14_H_9_N_2_O_2_Cl_2_F [M + H]^+^: 326.0019, found 325.9990.

*3,4-dichloro-1-(((E)-4-(p-tolyl)but-3-en-2-ylidene)amino)-1H-pyrrole-2,5-dione* (**15**). Pale yellow solid, mp 123–124 °C, yield = 65%. ^1^H-NMR (CDCl_3_) δ 7.22 (d, *J* = 7.8 Hz, 2H), 7.17 (d, *J* = 7.7 Hz, 2H), 6.08 (s, 1H), 5.47 (s, 1H), 2.56 (s, 3H), 2.33 (s, 3H); ^13^C-NMR (CDCl_3_) δ 149.6, 149.3, 139.4, 138.1, 137.3, 131.6, 129.7, 127.8, 111.8, 77.4, 77.2, 76.9, 29.8, 15.4; HRMS (ESI): *m*/*z* calcd for C_15_H_12_N_2_O_2_Cl_2_ [M + H]^+^: 345.0168, found 345.0170.

*3,4-dichloro-1-(((E)-4-(2,6-dichlorophenyl)but-3-en-2-ylidene)amino)-1H-pyrrole-2,5-dione* (**16**). Pale yellow solid, mp 212–213 °C, yield = 59%. ^1^H-NMR (CDCl_3_) δ 7.40 (d, *J* = 7.7 Hz, 1H), 7.28–7.19 (m, 2H), 6.97 (s, 1H), 5.34 (s, 1H), 2.53 (s, 3H); ^13^C-NMR (CDCl_3_) δ 149.5, 149.2, 139.8, 139.6, 137.2, 136.0, 133.6, 130.4, 129.2, 128.2, 107.0, 77.4, 77.2, 76.9, 15.3; HRMS (ESI): *m*/*z* calcd for C_14_H_8_N_2_O_2_Cl_4_ [M + H]^+^: 398.9232, found 398.9260.

### 3.4. Synthesis of (E)-2-(1-hydroxy-3-phenylallylidene)-4,5-dimethoxycyclopent-4-ene-1,3-dione (Linderone)

This compound was prepared according to literature [11]: yield = 84%. NMR data consistent with literature [11]: ^1^H-NMR (CDCl_3_) δ 11.56 (s, 1H), 7.67 (s, 2H), 7.62 (m, 2H), 7.41 (m, 3H), 4.22 (s, 3H), 4.17 (s, 3H).

### 3.5. Synthesis of (E)-4,5-dimethoxy-2-(1-methoxy-3-phenylallylidene)cyclopent-4-ene-1,3-dione (Methyllinderone)

Methyllinderone was prepared from linderone according to the literature [11]. Yield = 93%; ^1^H-NMR (CDCl_3_) δ 7.94 (d, *J* = 15.6 Hz, 1H), 7.55–7.65 (m, 2H), 7.52 (d, *J* = 15.6 Hz, 1H), 7.30–7.40 (m, 3H), 4.19 (s, 6H), 4.10 (s, 3H).

### 3.6. General Procedure for the Synthesis of Copound ***17***

*3,4-dichloro-1H-pyrrole-2,5-dione* (**17**). The compound **17** was prepared according to the literature [12]. Orange crystal powder, mp 178–179 °C, yield = 82%. ^1^H-NMR (500 MHz, DMSO-*d*_6_) δ 11.73 (s, 1H); ^13^C-NMR (126 MHz, DMSO-*d*_6_) δ 164.48, 133.26. HRMS (ESI): *m*/*z* calcd for C_4_HNO_2_Cl_2_ [M + H]^+^: 163.9312, found 163.9306.

### 3.7. Insecticidal Biological Assay

All bioassays were performed on representative test organisms reared in the laboratory. The bioassay was repeated at 25 ± 1 °C according to statistical requirements. Assessments were made on a dead/alive basis and mortality rates were corrected using Abbott’s formula [13]. Evaluations are based on a percentage scale of 0–100 in which 0 = no activity and 100 = total kill.

#### 3.7.1. Stomach Toxicity against Oriental Armyworm (*Mythimna separata*)

The stomach toxicities of compounds **1**–**8**, **10** against oriental armyworm were evaluated by foliar application using the reported procedure [14]. For the foliar armyworm tests, individual corn leaves were placed on moistened pieces of filter paper in Petri dishes. The leaves were then sprayed with the test solution and allowed to dry. The dishes were infested with 10 fourth-instar oriental armyworm larvae. Percentage mortalities were evaluated 3 days after treatment. Each treatment was performed three times.

#### 3.7.2. Toxicity against Mosquito (*Culex pipiens pallens*)

The toxicities of compounds **1**–**8**, **10** against mosquito were evaluated according to the reported procedure [15,16]. One milliliter of different concentrated dilutions of each compound was added to 99 mL of water to obtain different concentrations of tested solution. Then 20 fourth-instar mosquito larvae were put into the solution. Percentage mortalities were evaluated 8 days after treatment. Each test was performed in triplicate.

### 3.8. In Vitro Antifungal Bioassay

The antifungal activities were screened and evaluated by the poison plate technique [17]. All final compounds were dissolved in DMF (0.1 mL) before mixing with potato dextrose agar (PDA; 9.9 mL). The compounds were tested at a concentration of 50 μg·mL^−1^. All fungi were cultivated in PDA at 27 ± 1 °C for 4 days to make new mycelium for the identification of antifungal activity. Then, mycelia dishes of approximately 5 mm diameter were cut from the culture medium. A mycelium was obtained using a germ-free inoculation needle and inoculated in the middle of the PDA plate aseptically. The inoculated plates were incubated at 27 ± 1 °C for 5 days. DMF in sterile distilled water served as the negative control, whereas hymexazol served as the positive control. Each treatment condition consisted of three replicates. Radial growth of the fungal colonies was measured, and the data were statistically analyzed. Inhibitory effects of the test compounds in vitro on these fungi were calculated by the formula *I* (%) = [(*C* − *T*)/(*C* − 0.5)] × 100, where *C* represents the diameter of fungal growth on untreated PDA, *T* represents the diameter of fungi on treated PDA, and *I* represents the inhibition rate.

## 4. Conclusions

A series of novel *N*-amino-maleimide derivatives containing hydrazone group were designed and synthesized and their insecticidal and antifungal activities were evaluated and discussed. The results of bioassays indicated that some compounds (compounds **2**, **3** and **8**) possessed excellent activities against mosquito and compounds **2**, **3** and **6** exhibited considerable activities against oriental armyworm. In particular, some compounds exhibited better antifungal activities against fourteen phytogenic fungi than linderone and methyllinderone. This shows that using natural active substance as a first lead structure, designing and synthesizing its derivatives is a good choice for discovering active pesticide molecules. Because compound **12** exhibited best broad-spectrum antifungal activities, it makes sense to further explore its fungicidal mechanism, chitinase inhibitory activity in vitro and discover the fungicide candidate.

## Figures and Tables

**Scheme 1:**
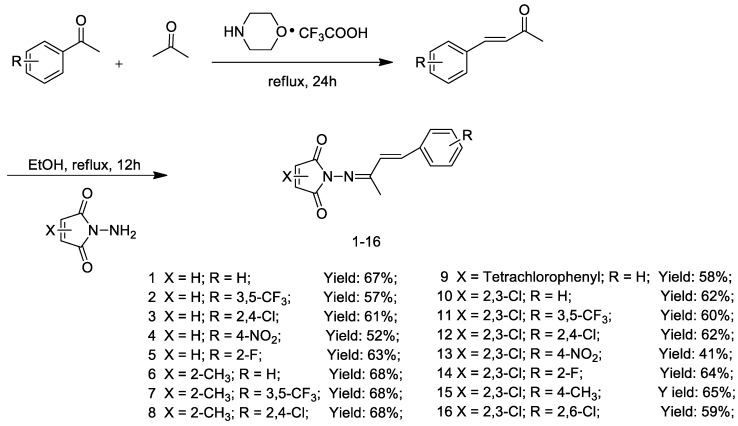
General synthetic route for compounds **1**–**16**.

**Figure 4:**
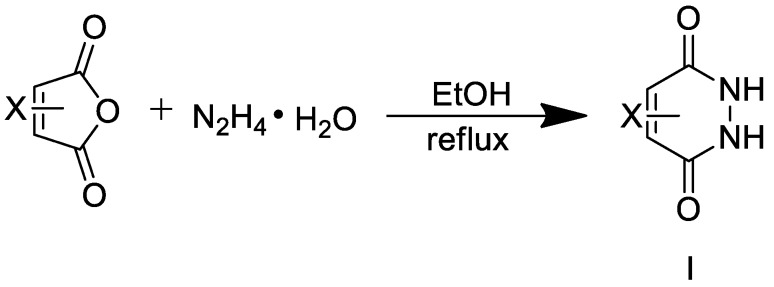
Synthesis of six-membered ring isomers.

**Table 1:** Larvacidal activities against oriental armyworm and mosquito of compounds **1**–**8**, **10**.

Compd.	Larvicidal Activity (%) at Concn (µg·mL^−1^)						
	Oriental Armyworm	Mosquito					
	600	10	5	2	1	0.5	0.25
**1**	25	50	- ^a^	-	-	-	-
**2**	60	100	100	100	100	100	60
**3**	50	100	100	100	100	100	60
**4**	10	40	-	-	-	-	-
**5**	25	30	-	-	-	-	-
**6**	65	50	-	-	-	-	-
**7**	40	100	20	-	-	-	-
**8**	5	100	100	100	100	100	20
**10**	20	100	100	40	-	-	-

*^a^* - no test data.

**Table 2:** Fungicidal activity of title compounds **1**–**16** and *Linderone*, *Methyllinderone* at 50 μg·mL^−1^.

Compd.	Inhibition Rate (%)													
	*A.S* *^a^*	*G.Z*	*P.I*	*S.S*	*B.C*	*R.S*	*F.O*	*C.A*	*B.B*	*R.C*	*H.M*	*C.L*	*F.M*	*P.C*
**1**	25.0	17.2	17.6	8.9	15.4	6.2	11.6	36.7	26.8	34.9	32.7	10.0	18.2	-*^b^*
**2**	33.3	20.7	23.5	37.5	34.6	54.3	23.3	26.7	66.1	50.0	32.7	22.5	30.3	-
**3**	8.3	24.1	17.6	17.9	26.9	37.0	32.6	46.7	35.7	57.0	34.7	32.5	57.6	-
**4**	25	37.9	29.4	26.8	34.6	18.5	27.9	46.7	89.3	29.1	61.2	27.5	39.4	-
**5**	8.3	10.3	17.6	8.9	7.7	12.3	14.0	36.7	25.0	31.4	30.6	5.0	15.2	-
**6**	25	13.8	11.8	32.1	19.2	30.9	16.3	13.3	62.5	23.3	20.4	15.0	30.3	-
**7**	16.7	10.3	23.5	21.4	19.2	53.1	20.9	36.7	51.8	58.1	26.5	22.5	30.3	-
**8**	16.7	17.2	17.6	39.3	23.1	45.7	25.6	56.7	48.2	64.0	34.7	32.5	27.3	-
**9**	33.3	44.8	23.5	5.4	3.8	14.8	16.3	30.0	7.1	40.7	26.5	17.5	21.2	-
**10**	41.7	44.8	58.8	75.0	73.1	66.7	39.5	46.7	75.0	79.1	44.9	47.5	48.5	-
**11**	27.8	39.5	18.2	63.4	52.4	43.2	45.7	50.0	14.3	89.1	47.2	40.7	46.2	33.3
**12**	44.4	47.4	50.0	87.5	71.4	67.9	65.7	65.0	42.9	98.2	63.9	70.4	61.5	55.6
**13**	44.4	36.8	27.3	26.8	47.6	42	51.4	35.0	34.7	61.8	27.8	18.5	38.5	27.8
**14**	44.4	39.5	54.5	60.6	47.6	67.9	45.7	25.0	67.3	100.0	55.6	44.4	46.2	69.4
**15**	44.4	44.7	63.6	66.2	52.4	63.0	45.7	50.0	36.7	90.9	52.8	51.9	46.2	69.4
**16**	44.4	42.1	45.5	63.4	52.4	61.7	45.7	55.0	51.0	89.1	52.8	37.0	53.8	63.9
*Linderone*	27.8	31.6	27.3	66.2	47.6	28.4	51.4	30.0	57.1	74.5	27.8	33.3	26.9	27.8
*Methyllinderon*	38.9	31.6	27.3	42.3	42.9	24.7	34.3	20.0	8.2	58.2	38.9	33.3	38.5	27.8
*carbendazim*	<50	100	100	100	<50	100	<50	<50	<50	100	100	100	<50	<50
*chlorothalonil*	73.3	<50	86.4	<50	100	100	100	73.3	100	100	91.3	91.3	100	100

*^a^ A.S*: *Alternaria solani*; *G.Z*: *Gibberella zeae*; *P.I*: *Phytophthora infestans*; *S.S*: *Sclerotinia sclerotiorum*; *B.C*: *Botrytis cinereal*; *R.S*: *Rhizoctonia solani*; *F.O*: *Fusarium oxysporum* f.sp. *cucumerinum*; *C.A*: *Cercospora arachidicola*; *B.B*: *Botryosphaeria berengeriana*; *R.C*: *Rhizoctonia cerealis*; *H.M*: *Helminthosporium maydis*; *C.L*: *Colleetotrichum lagenarium*; *F.M*: *Fusarium moniliforme*; *P.C*: *Phytophthora capsica*. *^b^* - no test data.

**Table 3:** Fungicidal activity of compounds **12**, **14**, **17** against *S. sclerotiorum* and *R. cerealis.*

Compd.	Inhibition Rate (%)			
	*S. sclerotiorum*	*R. cerealis*		
	50 μg·mL^−1^	50 μg·mL^−1^	25 μg·mL^−1^	12.5 μg·mL^−1^
**12**	- ^a^	-	95.8	60.6
**14**	-	-	77.5	47.9
**17**	21.4	23.6	-	-

*^a^* - no test data.

## Supplementary Materials

The following are available online, Figures S1–S34, NMR spectral information of compounds **1**–**17**.
