# Supplementary material for: Natural Product-Based Pesticide Discovery: Design, Synthesis and Bioactivity Studies of N-Amino-Maleimide Derivatives
Source: Molecules. 2018 Jun 24;23(7):1521. doi: 10.3390/molecules23071521 (PMC6099656; doi:10.3390/molecules23071521)
Supplement: Supplementary file 1 [file molecules-23-01521-s001.pdf]

Electronic Supporting Information (ESI) for:

Design, Synthesis and Bioactivity Studies of N-Amino-Maleimide Derivatives Containing Hydrazone Group

Content

^{1}H-NMR and ^{13}C-NMR Spectra (Figure S1-S34)

for compounds **1-17**

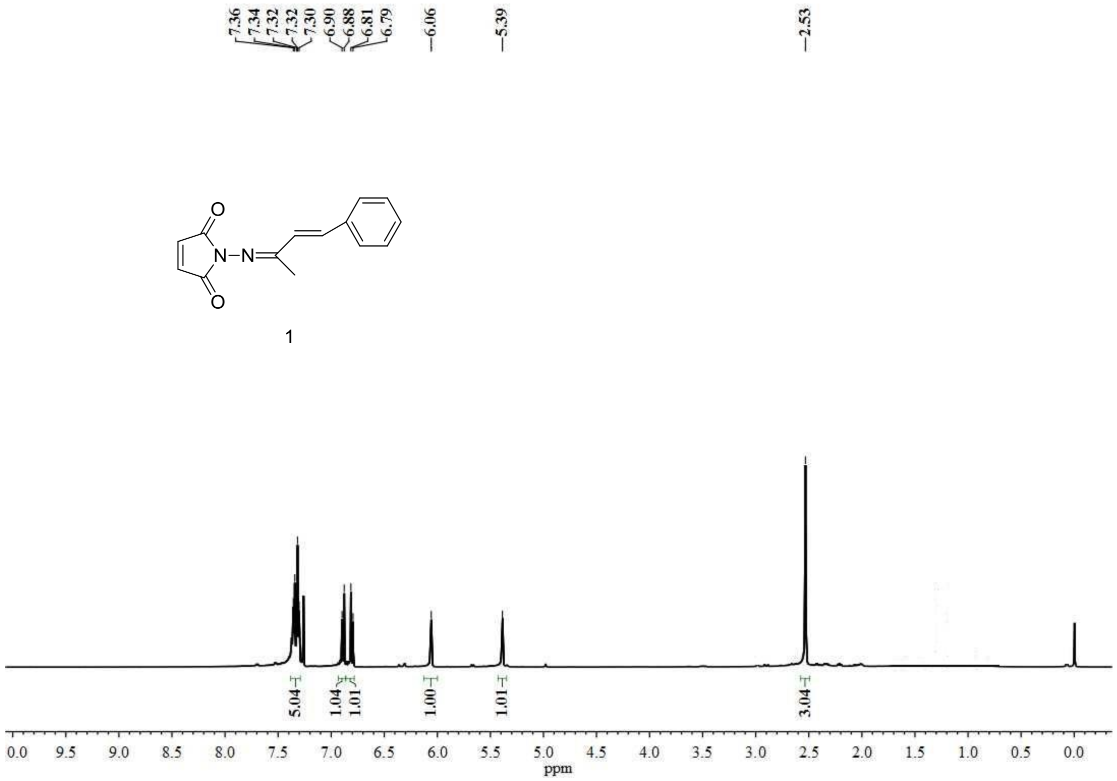

Figure S1 ^{1}H NMR spectra of compound 1

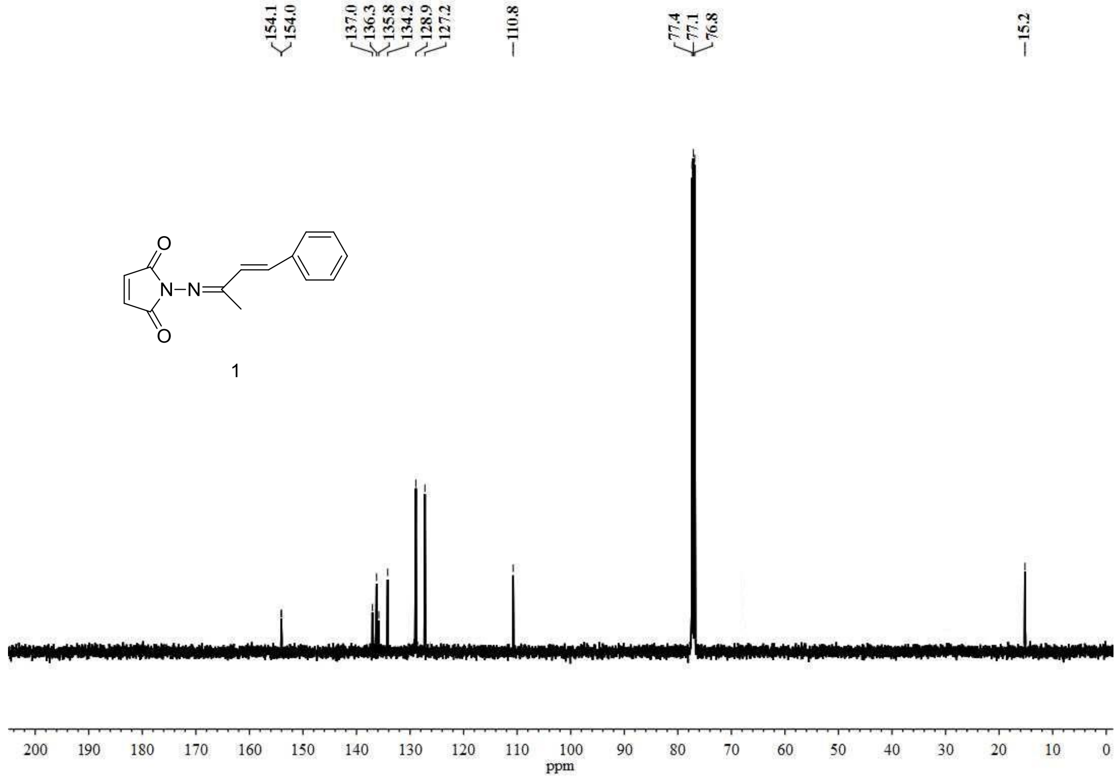

Figure S2 ^{13}C NMR spectra of compound 1

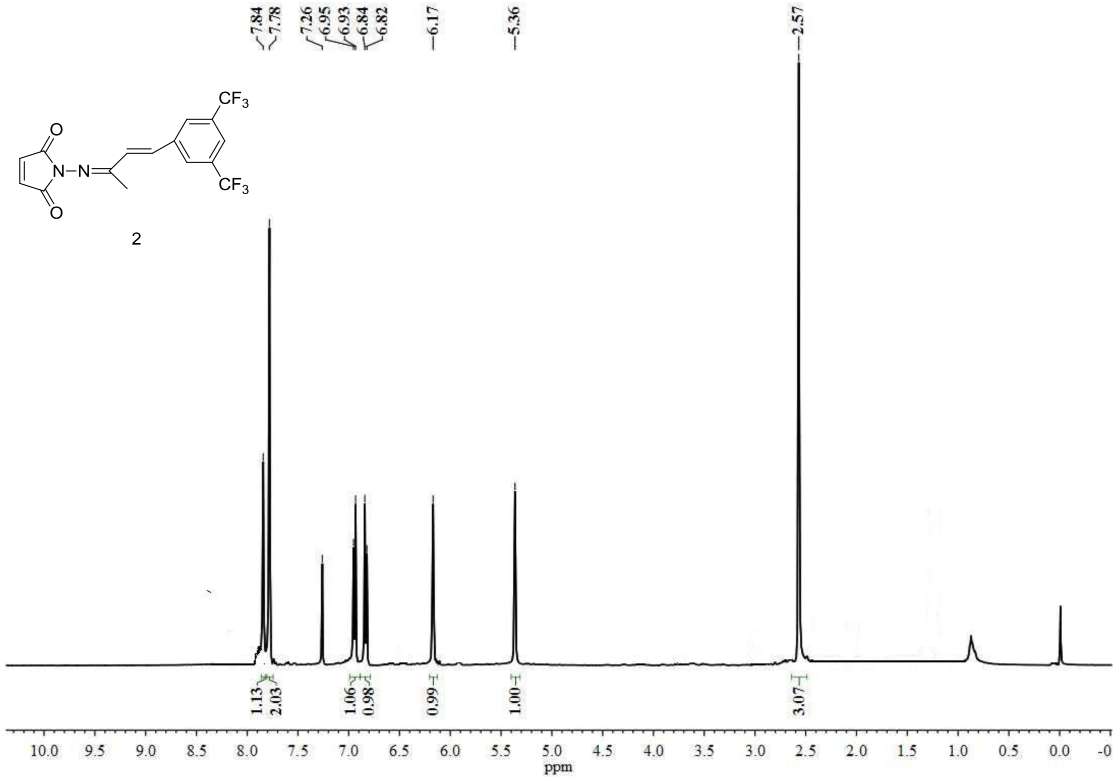

Figure S3 ^{1}H NMR spectra of compound 2

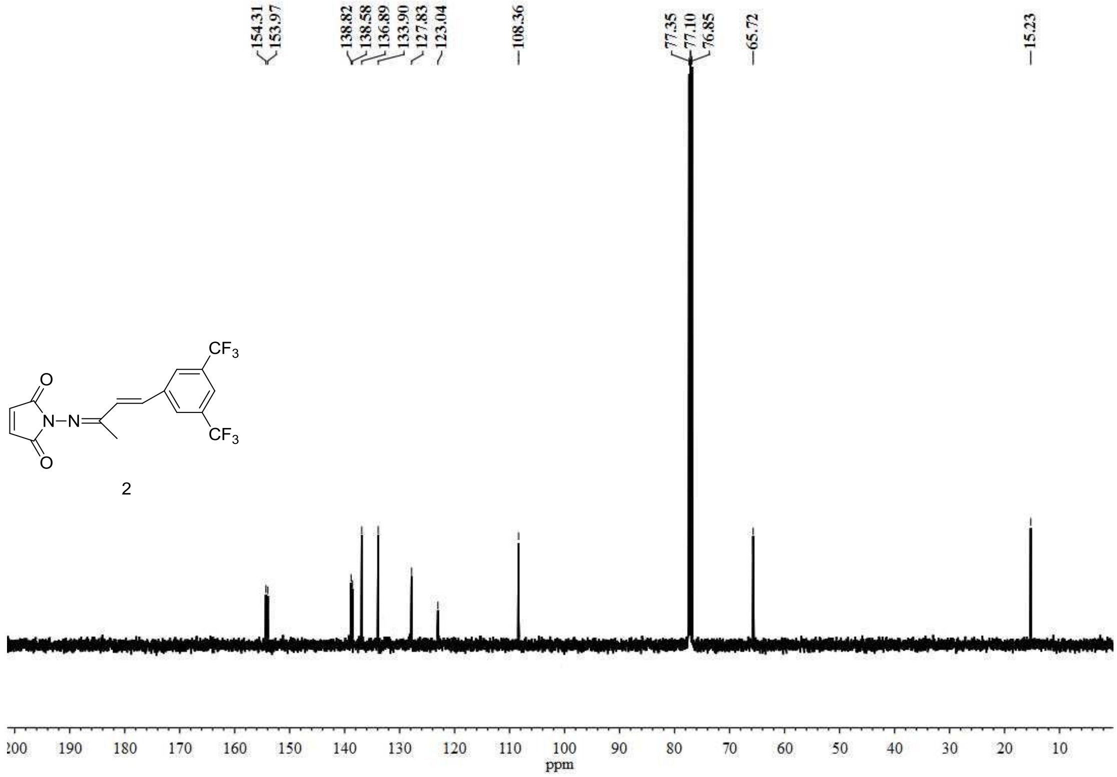

Figure S4 ^{13}C NMR spectra of compound 2

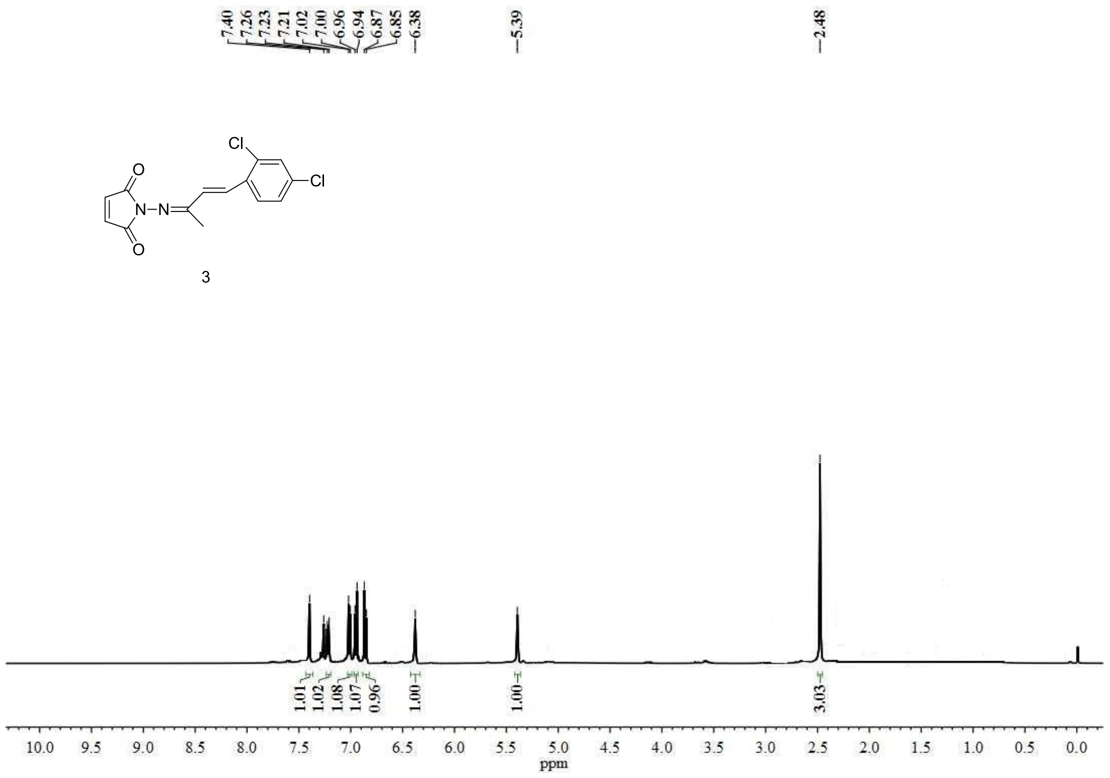

Figure S5 ^{1}H NMR spectra of compound 3

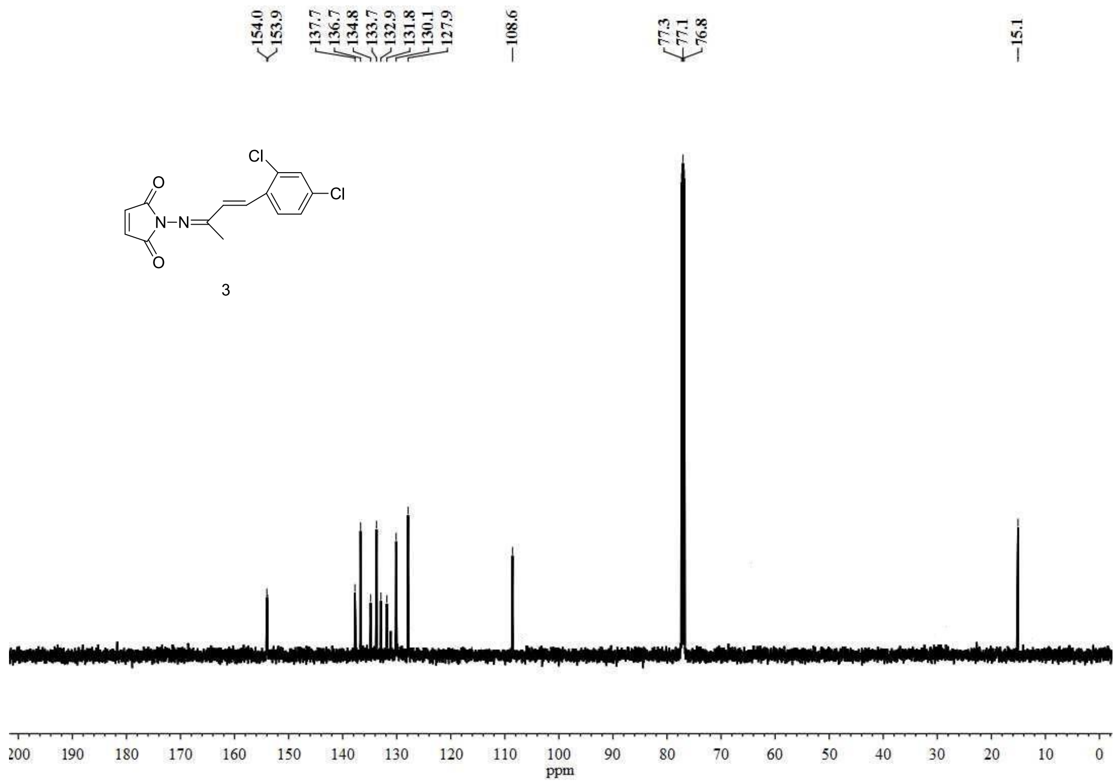

Figure S6 ^{13}C NMR spectra of compound 3

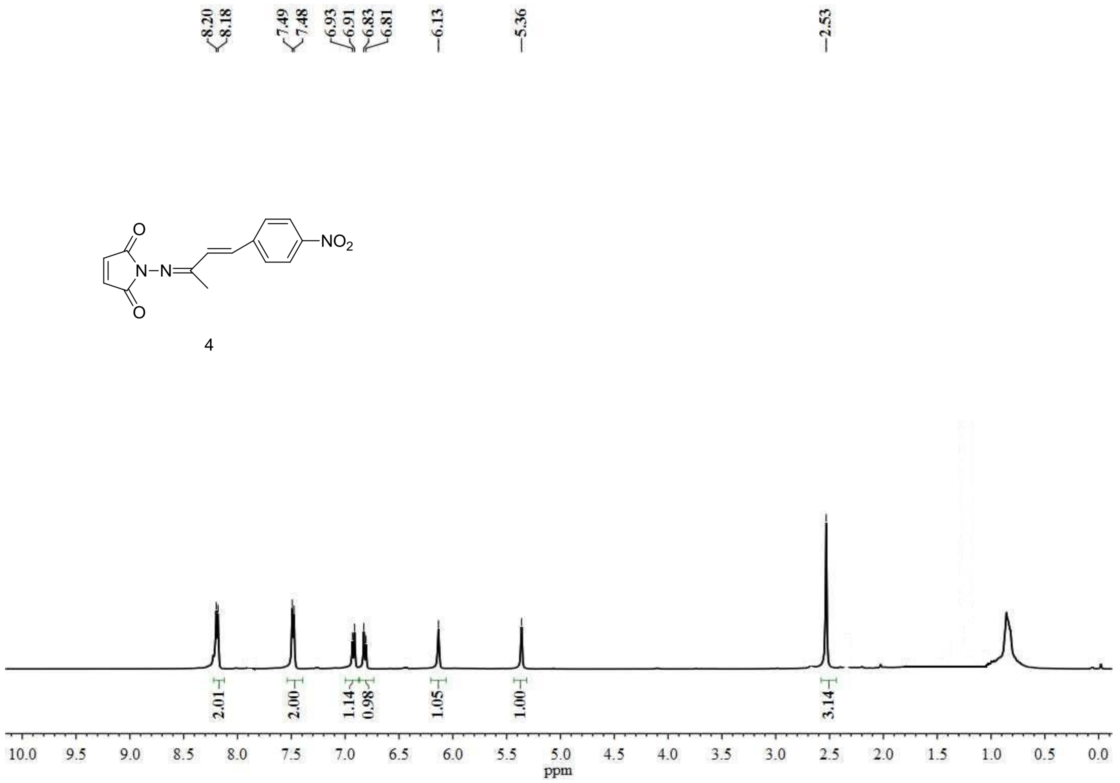

Figure S7 ^{1}H NMR spectra of compound 4

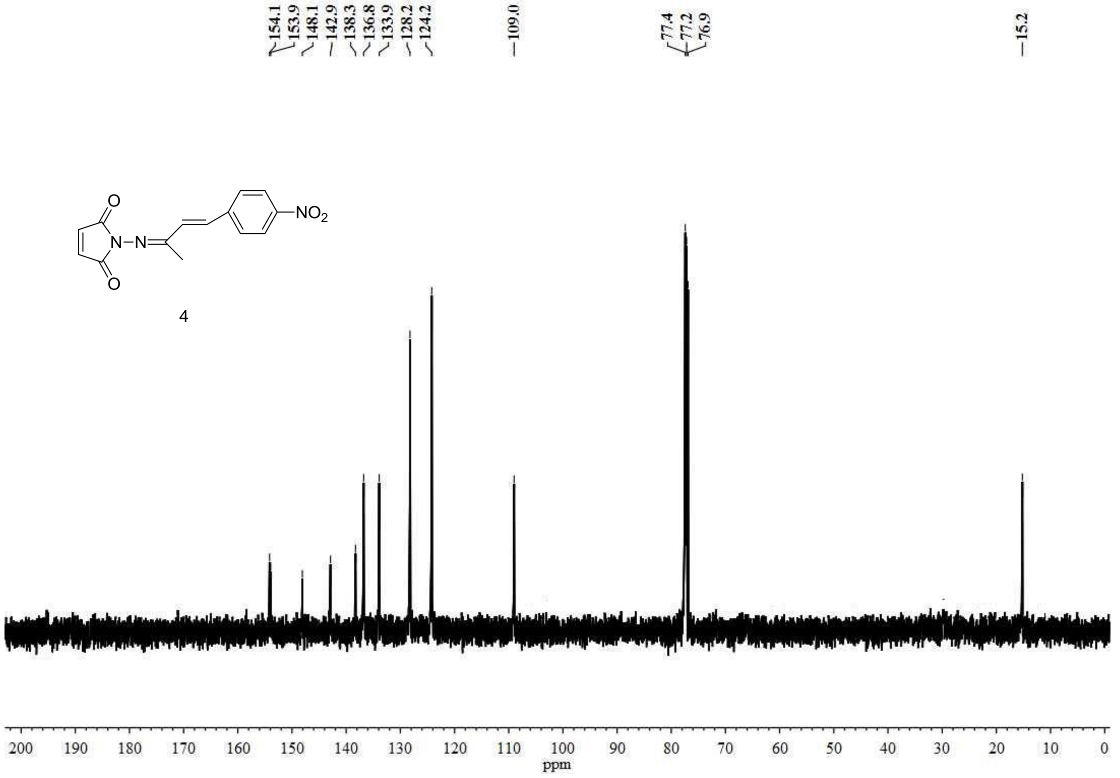

Figure S8 ^{13}C NMR spectra of compound 4

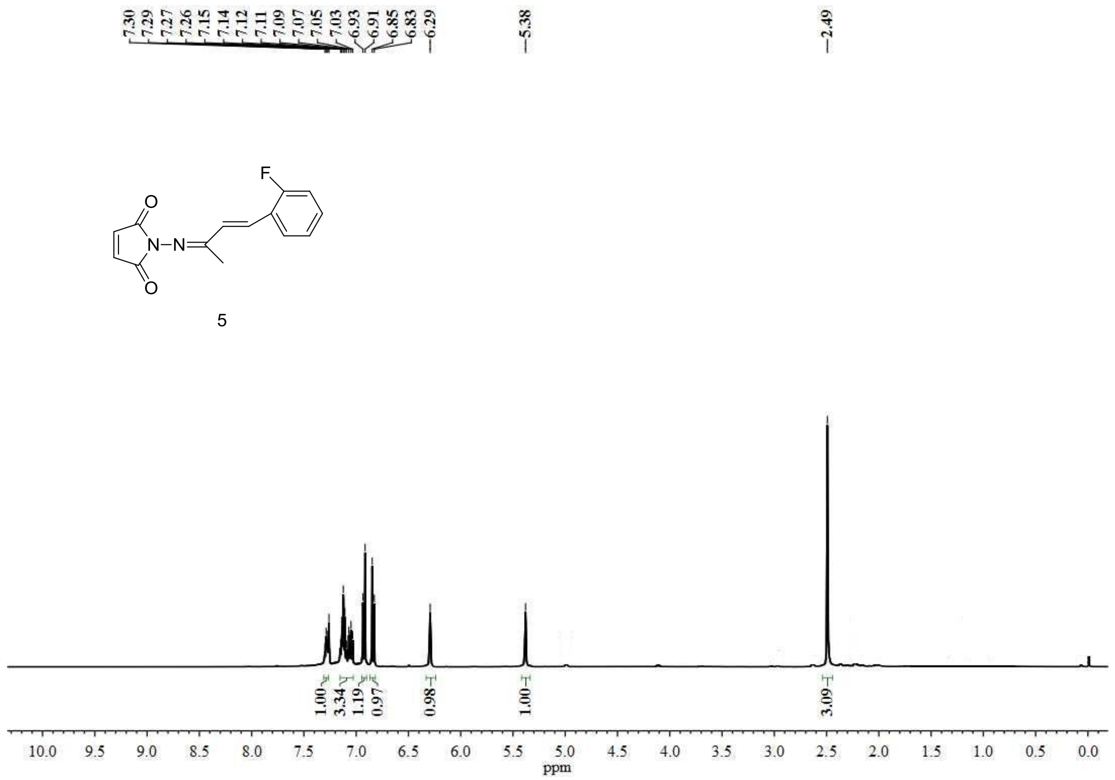

Figure S9 ^{1}H NMR spectra of compound 5

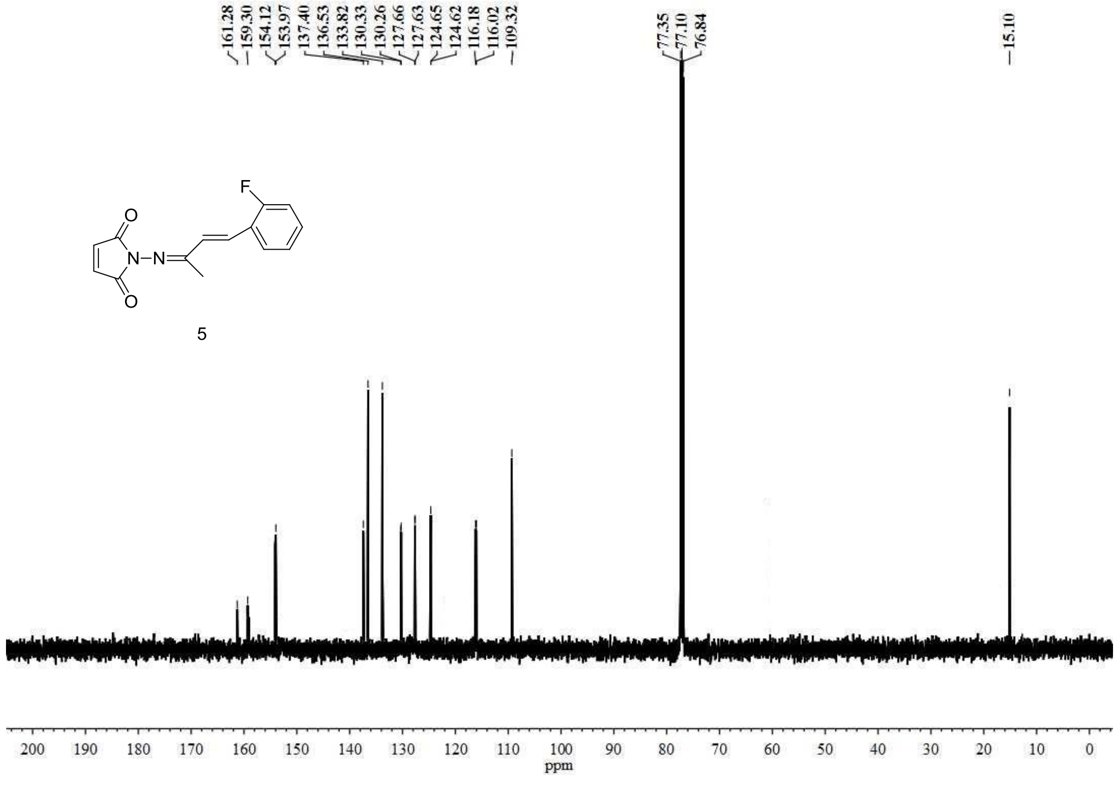

Figure S10 ^{13}C NMR spectra of compound 5

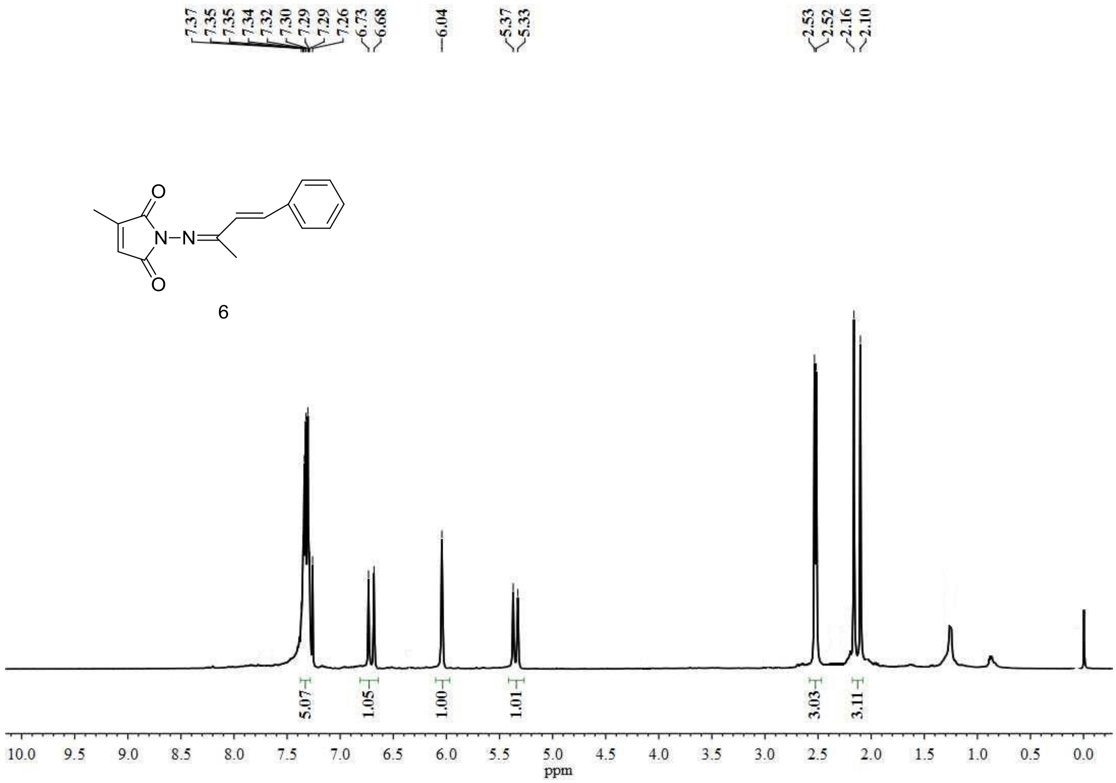

Figure S11 ^{1}H NMR spectra of compound 6

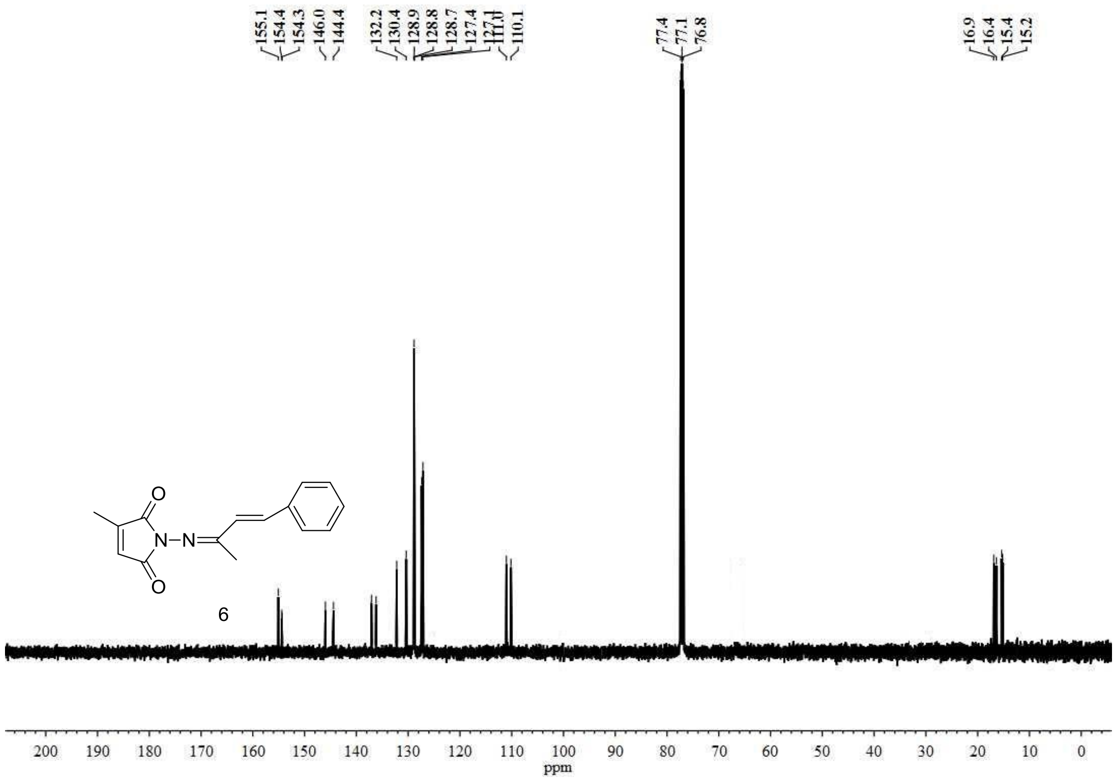

Figure S12 ^{13}C NMR spectra of compound 6

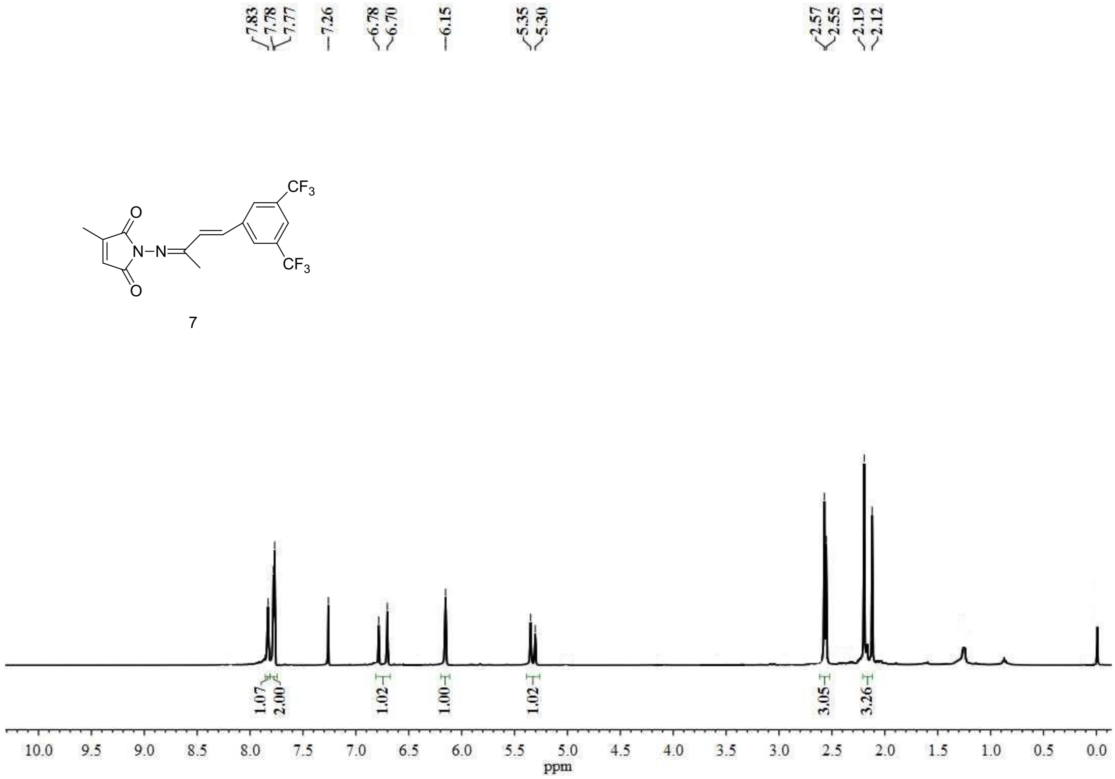

Figure S13 ^{1}H NMR spectra of compound 7

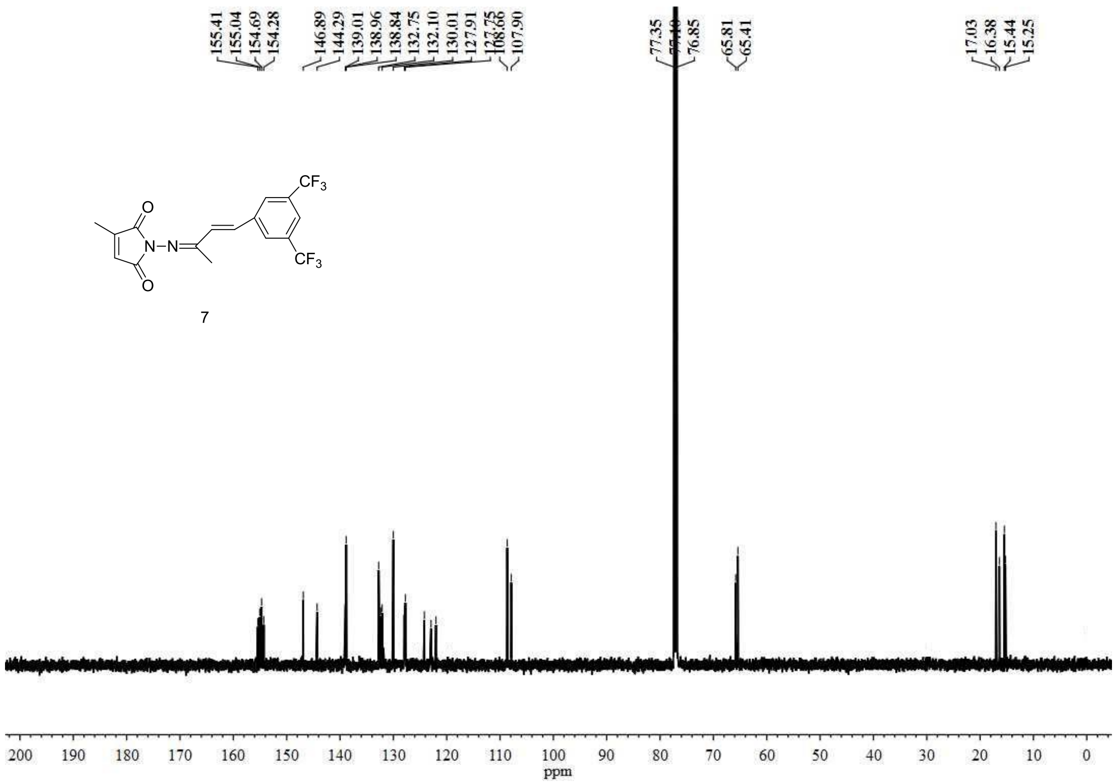

Figure S14 ^{13}C NMR spectra of compound 7

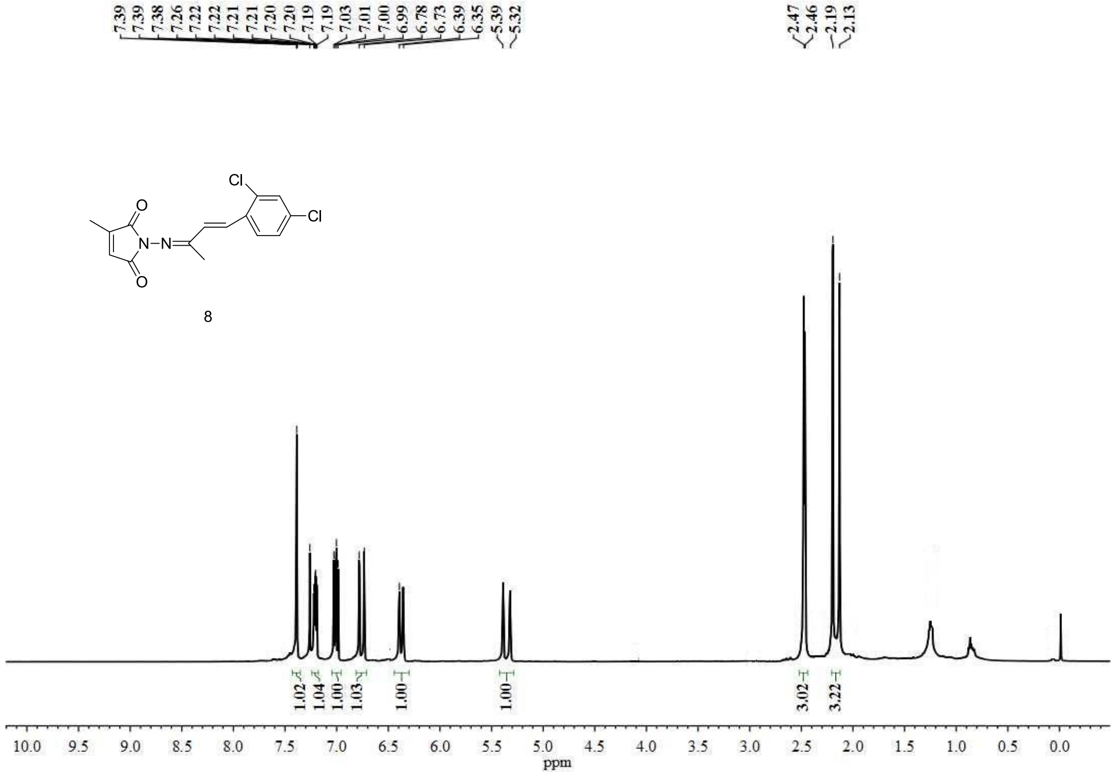

Figure S15 ^{1}H NMR spectra of compound 8

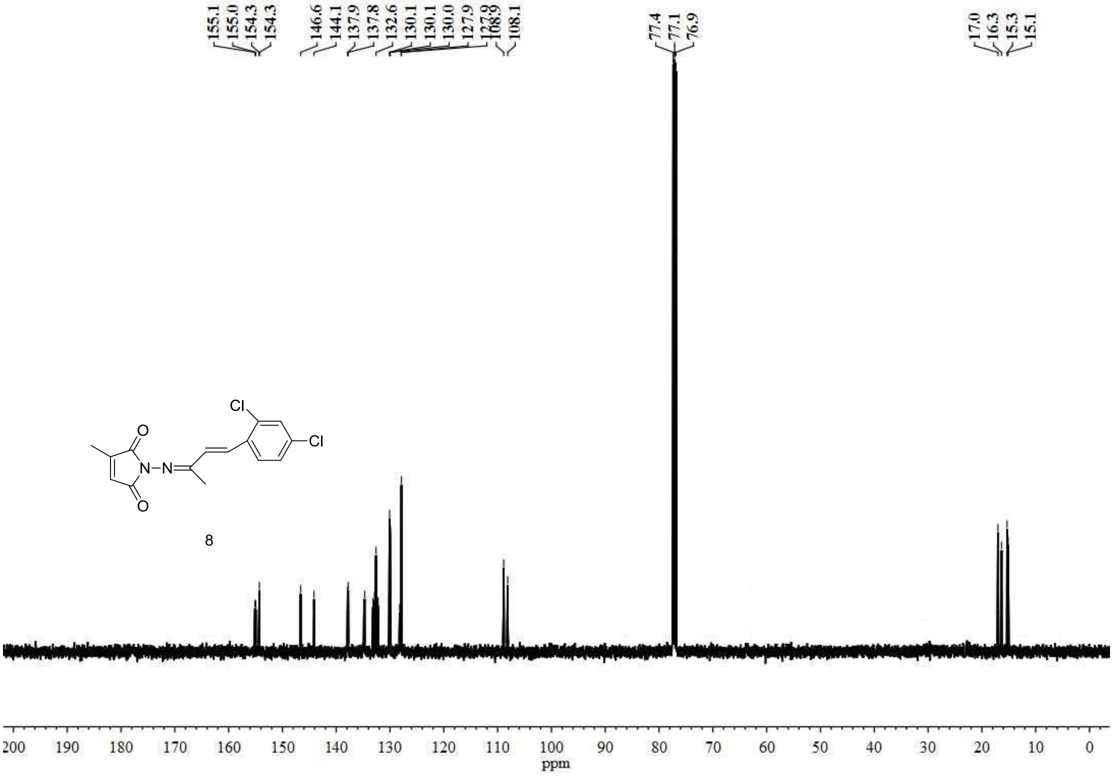

Figure S16 ^{13}C NMR spectra of compound 8

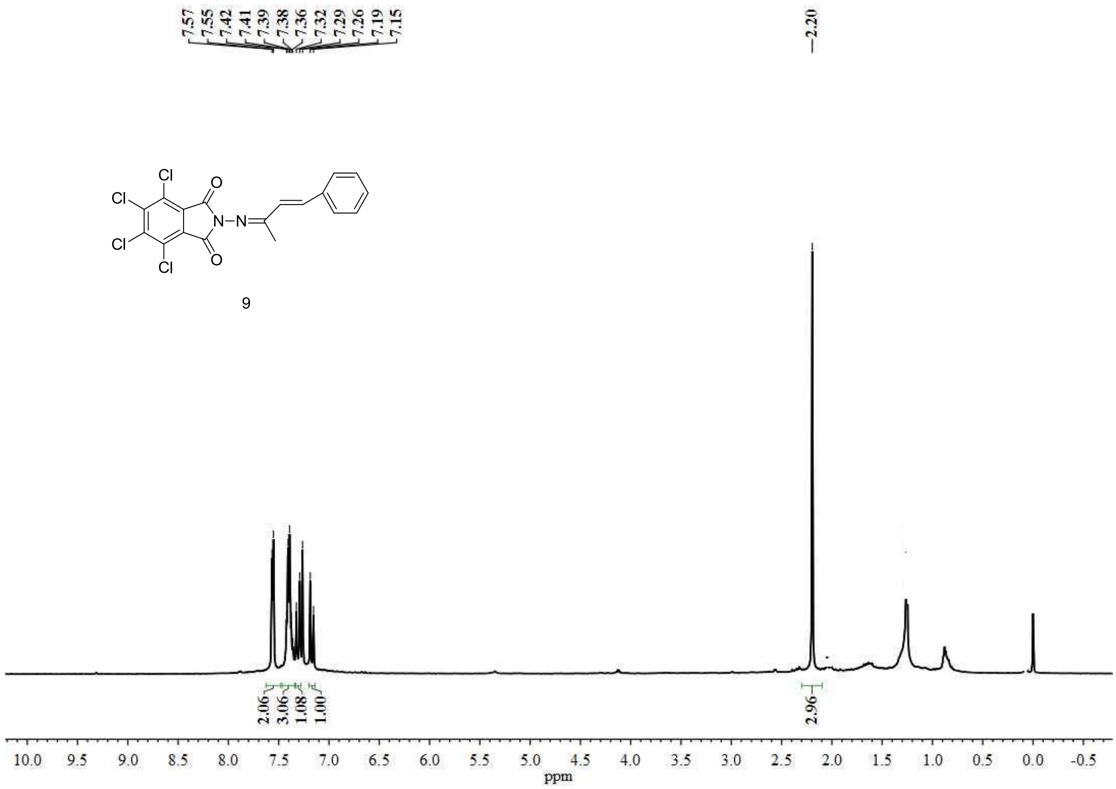

Figure S17 ^{1}H NMR spectra of compound 9

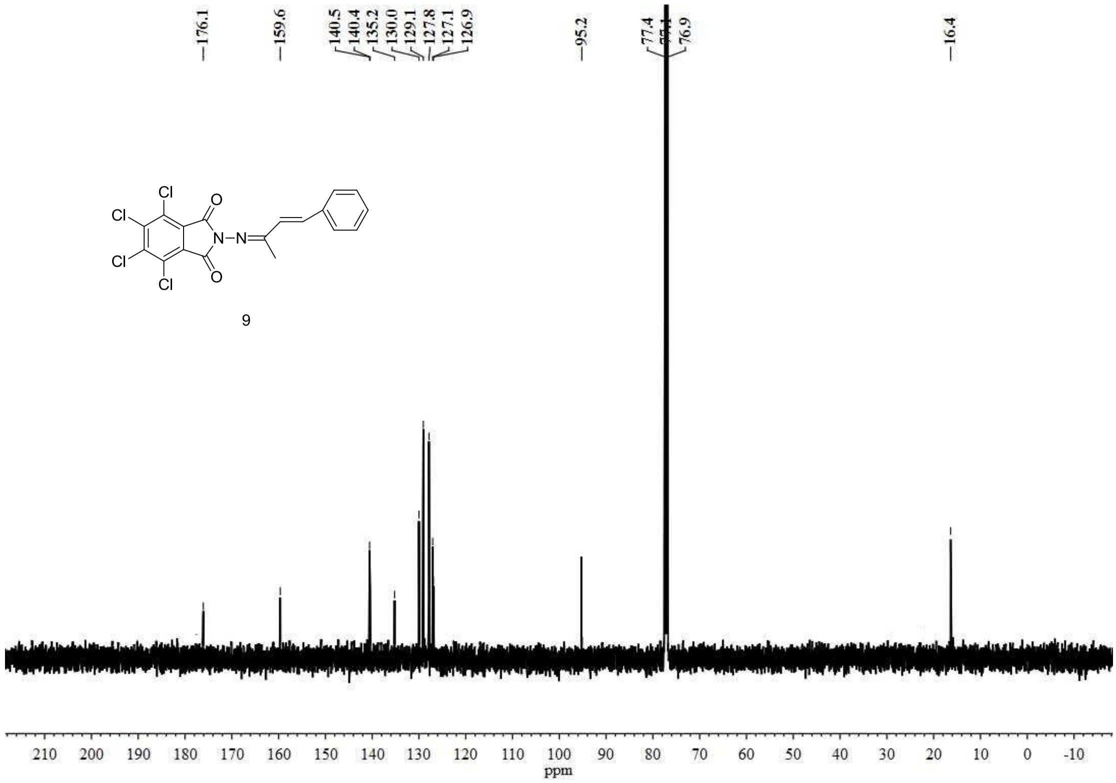

Figure S18 ^{13}C NMR spectra of compound 9

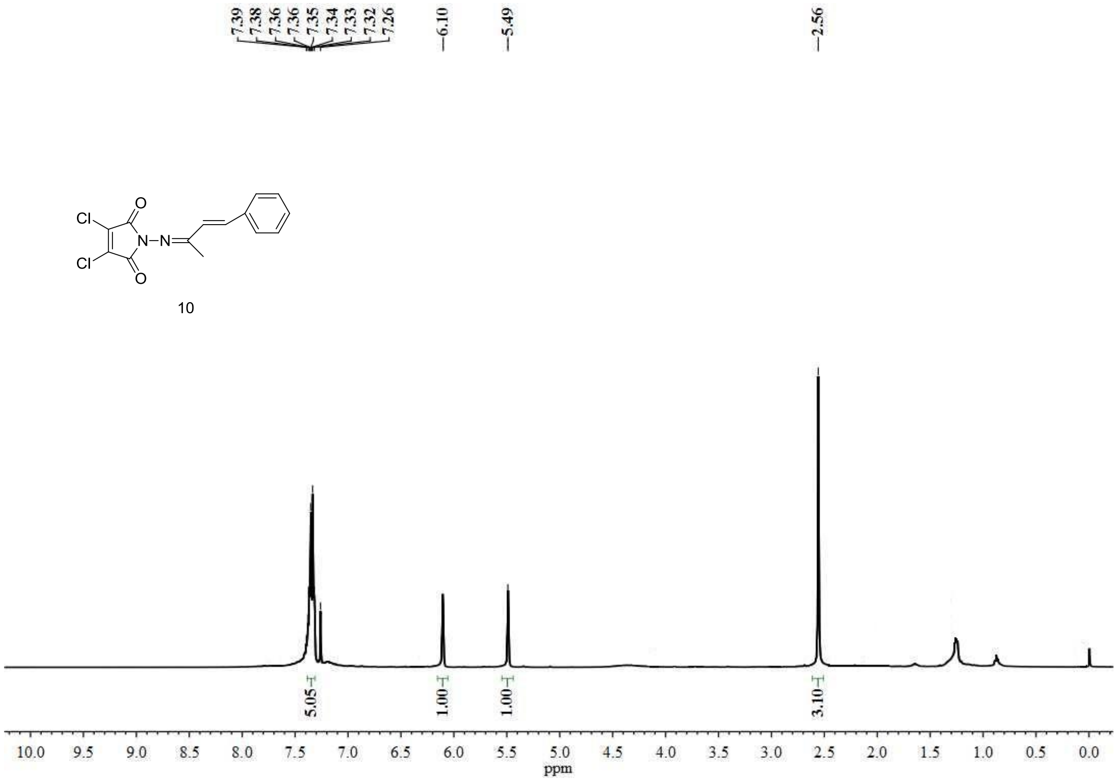

Figure S19 ^{1}H NMR spectra of compound 10

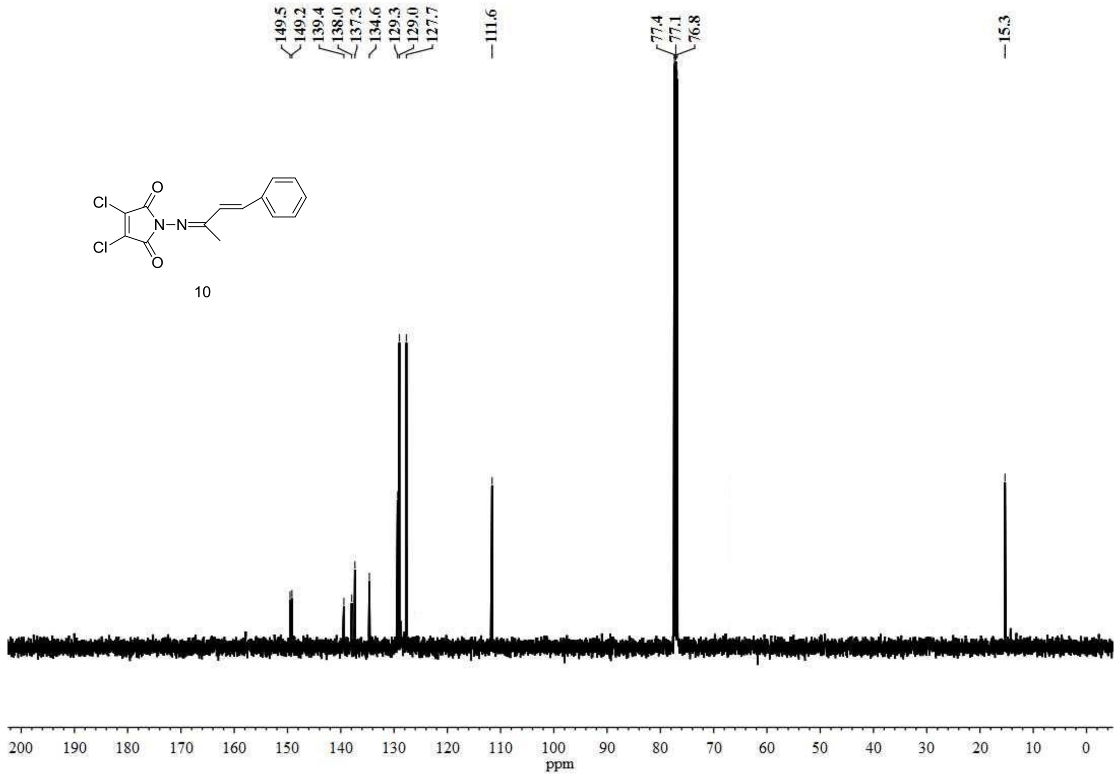

Figure S20 ^{13}C NMR spectra of compound 10

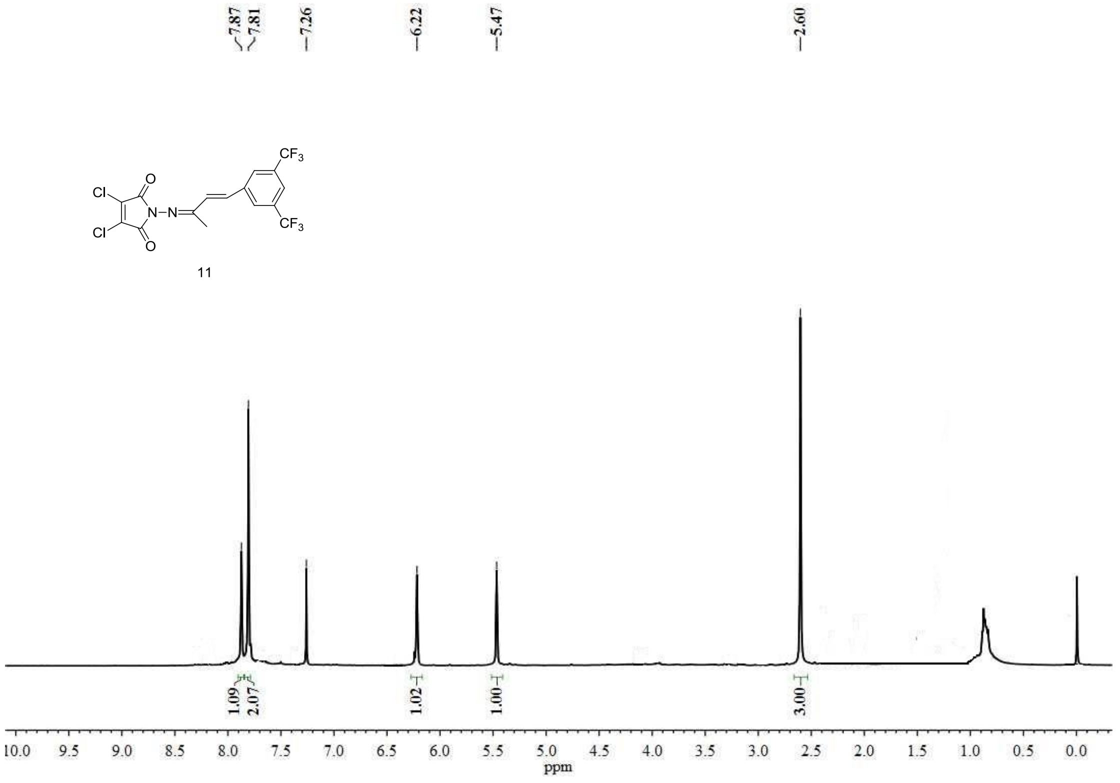

Figure S21 ^{1}H NMR spectra of compound 11

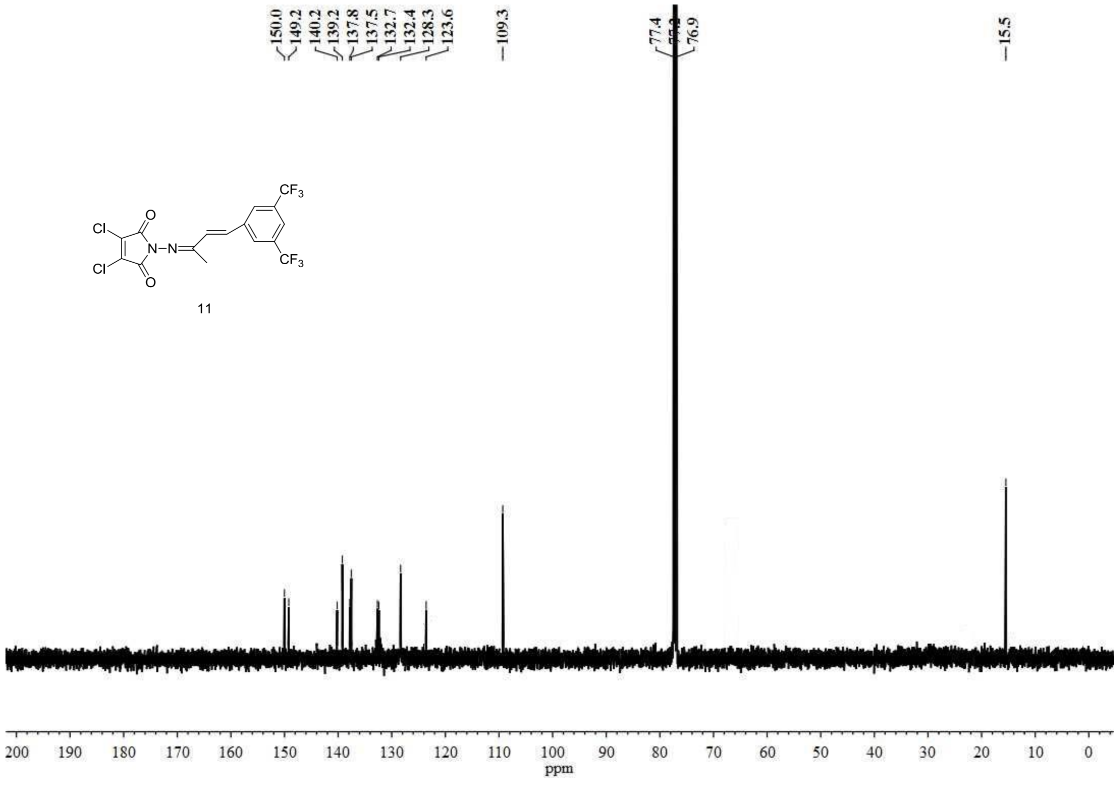

Figure S22 ^{13}C NMR spectra of compound 11

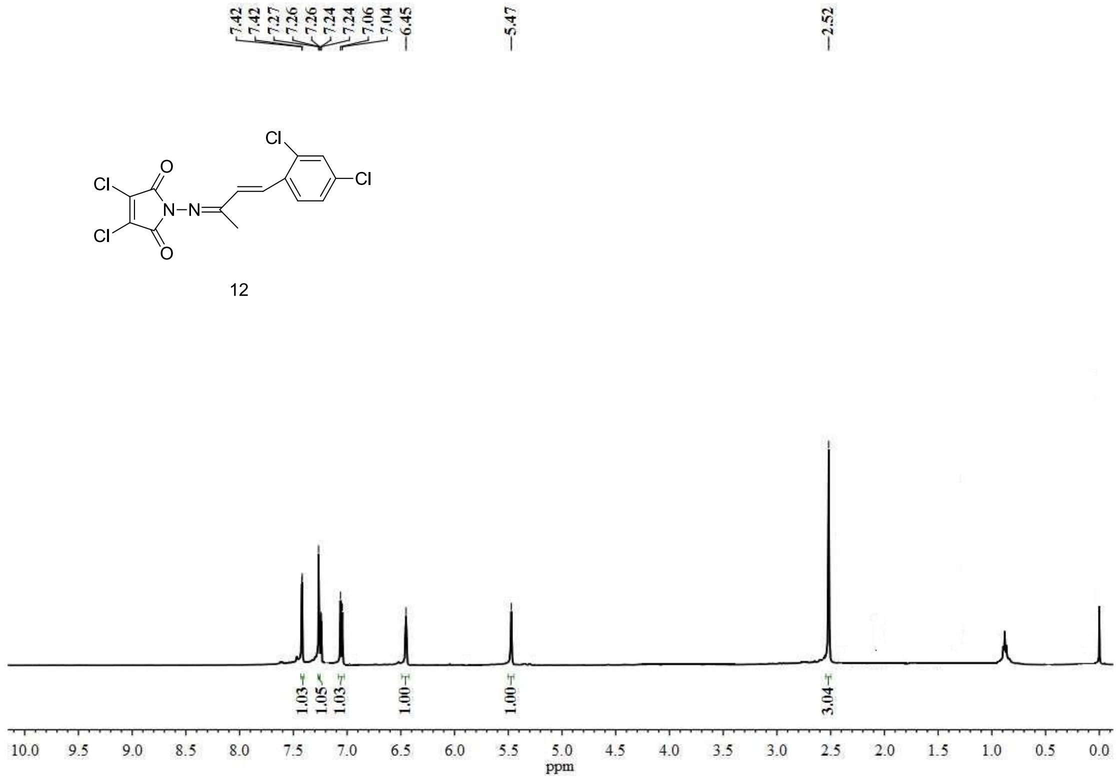

Figure S23 ^{1}H NMR spectra of compound 12

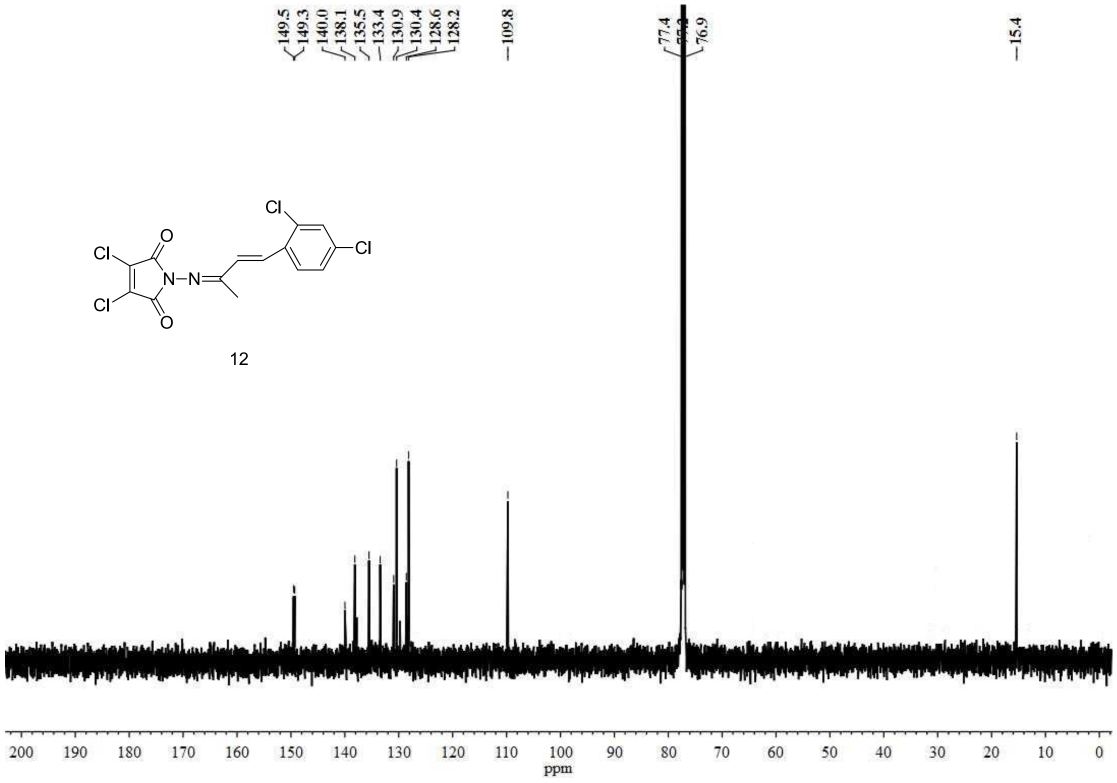

Figure S24 ^{13}C NMR spectra of compound 12

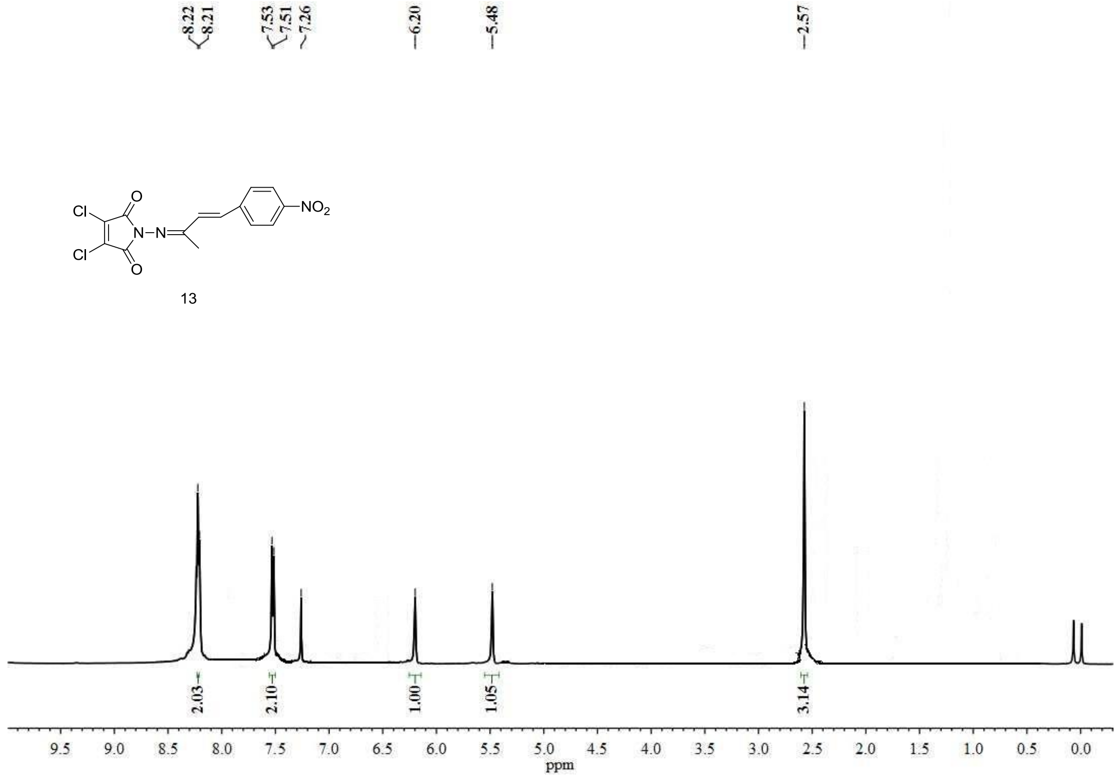

Figure S25 ^{1}H NMR spectra of compound 13

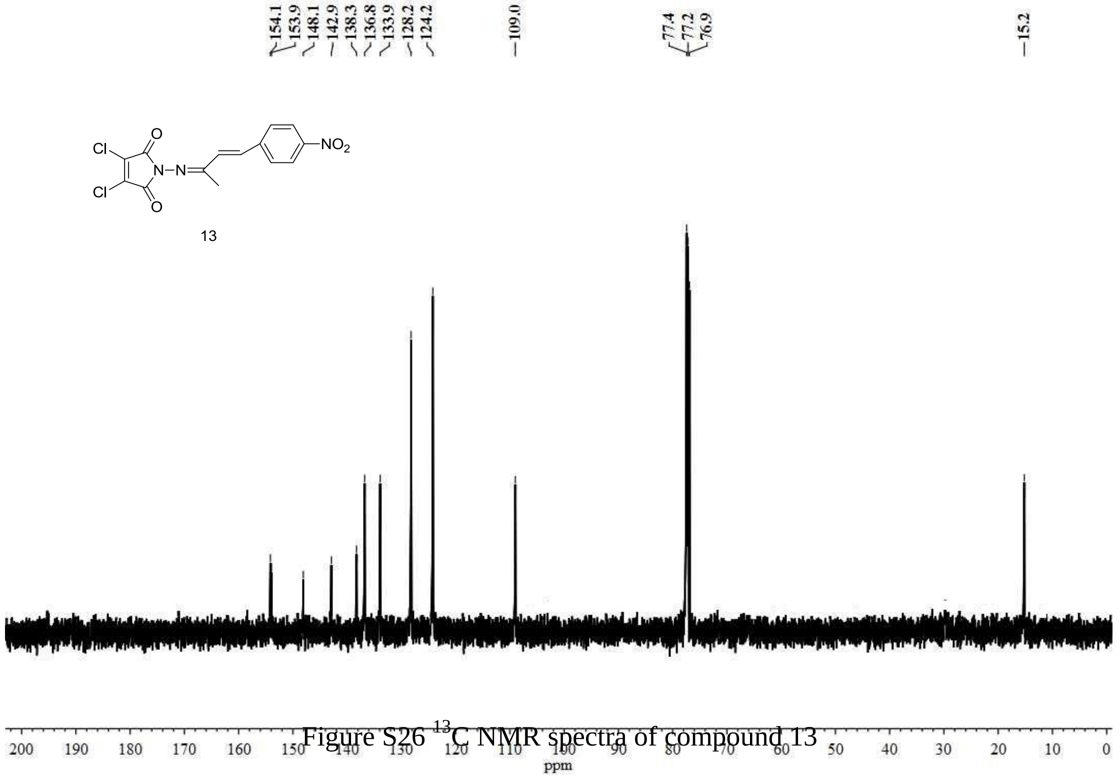

Figure S26 ^{13}C NMR spectra of compound 13

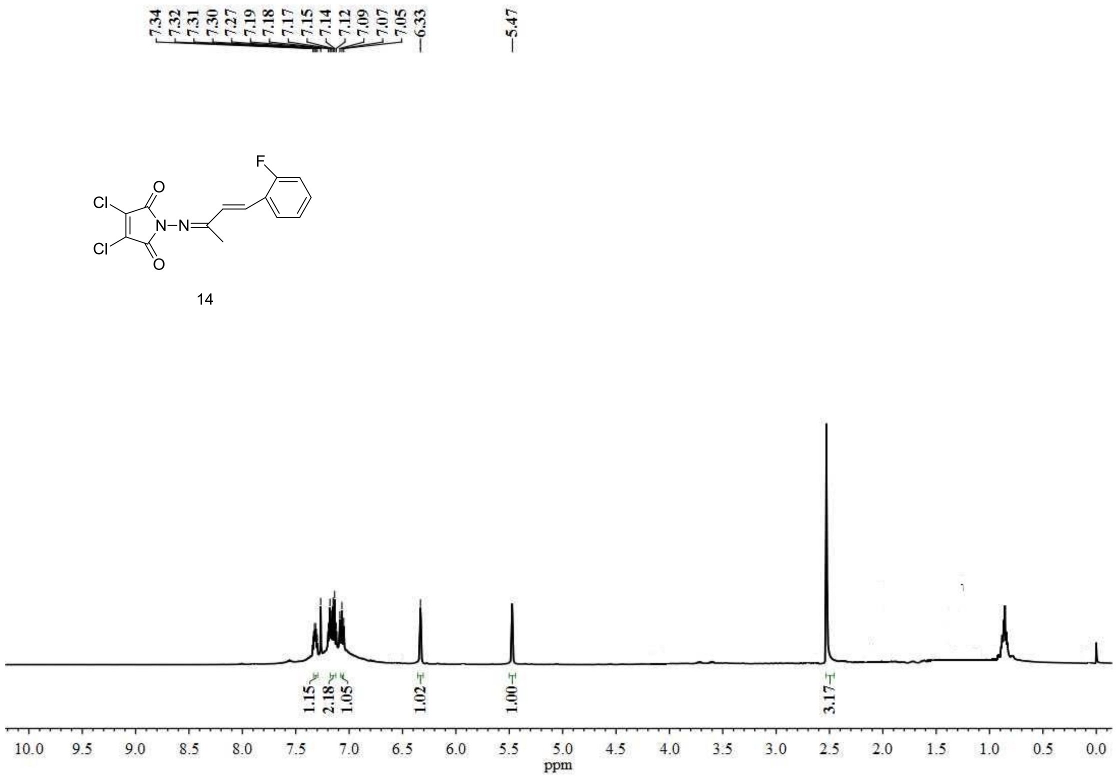

Figure S27 ^{1}H NMR spectra of compound 14

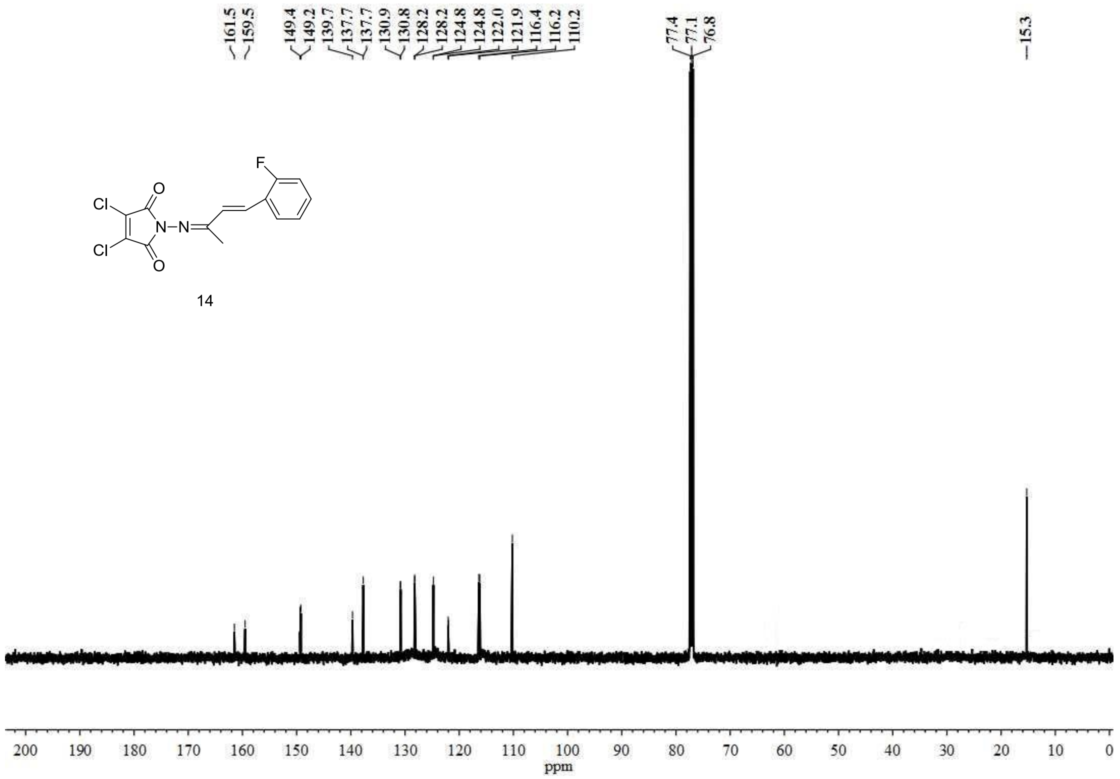

Figure S28 ^{13}C NMR spectra of compound 14

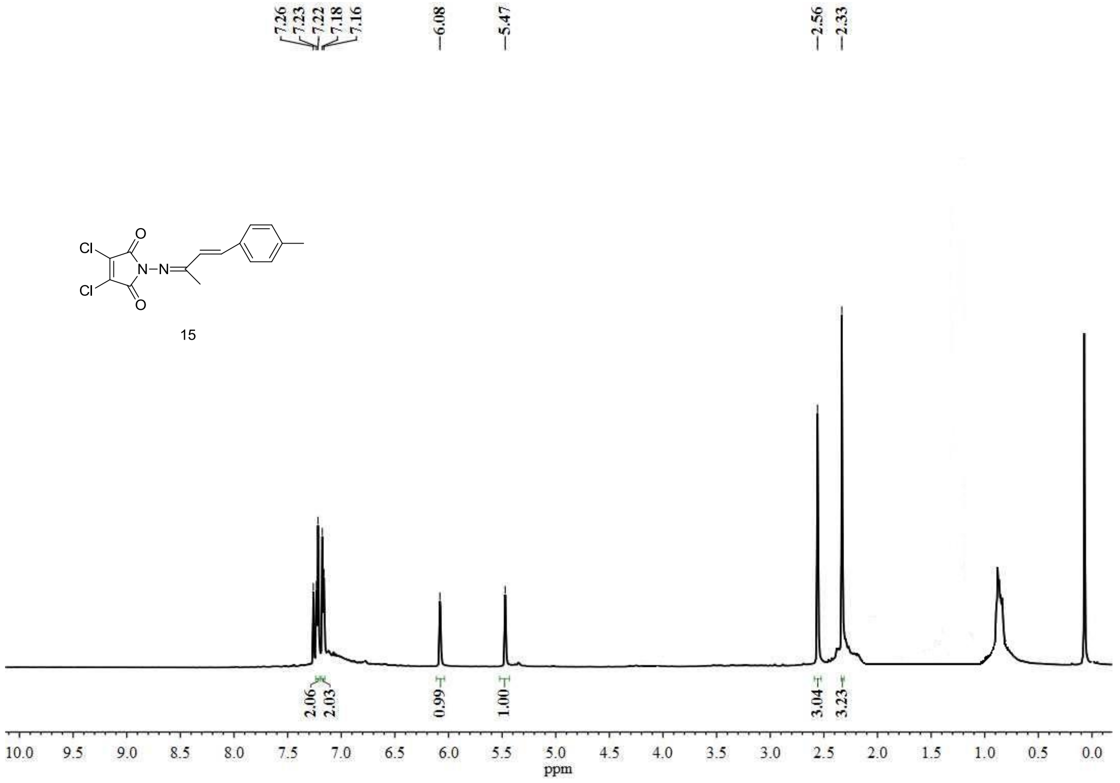

Figure S29 ^{1}H NMR spectra of compound 15

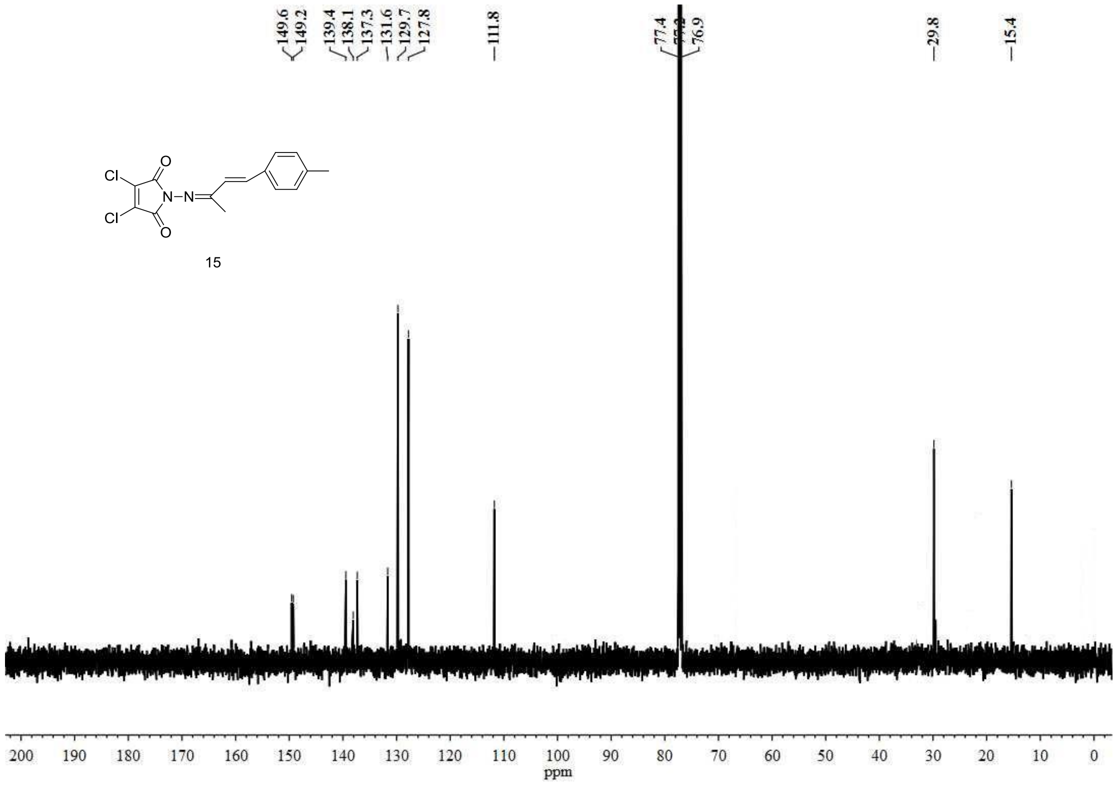

Figure S30 ^{13}C NMR spectra of compound 15

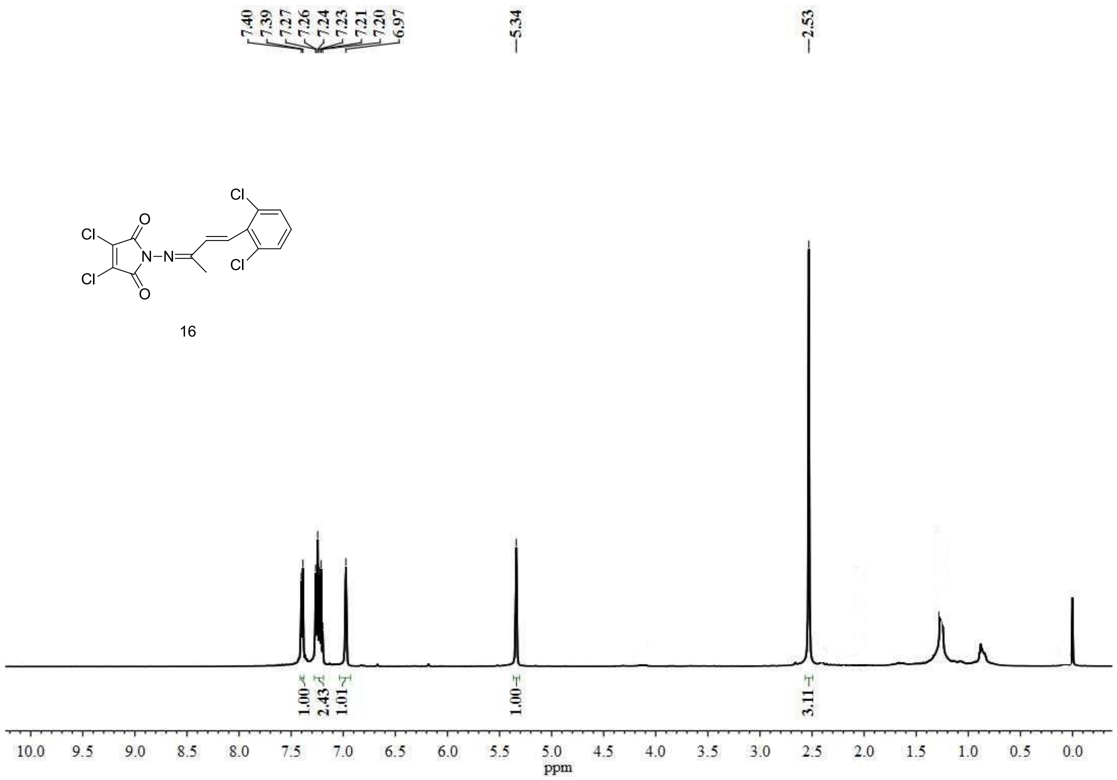

Figure S31 ^{1}H NMR spectra of compound 16

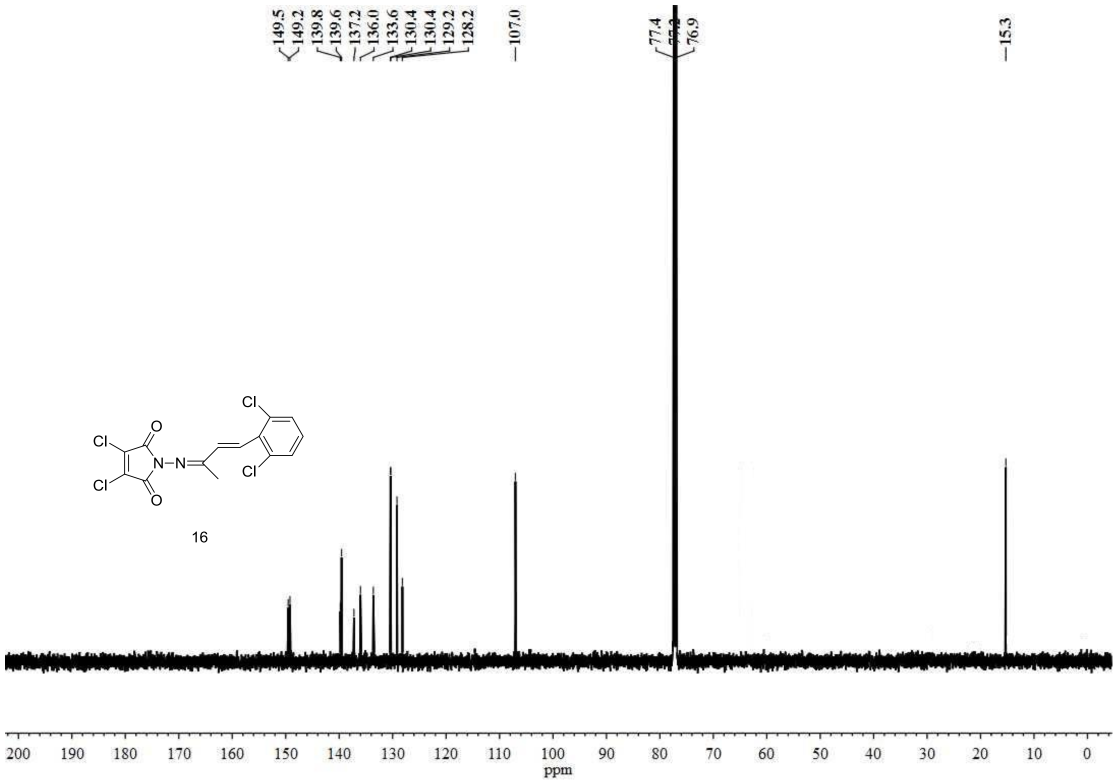

Figure S32 ^{13}C NMR spectra of compound 16

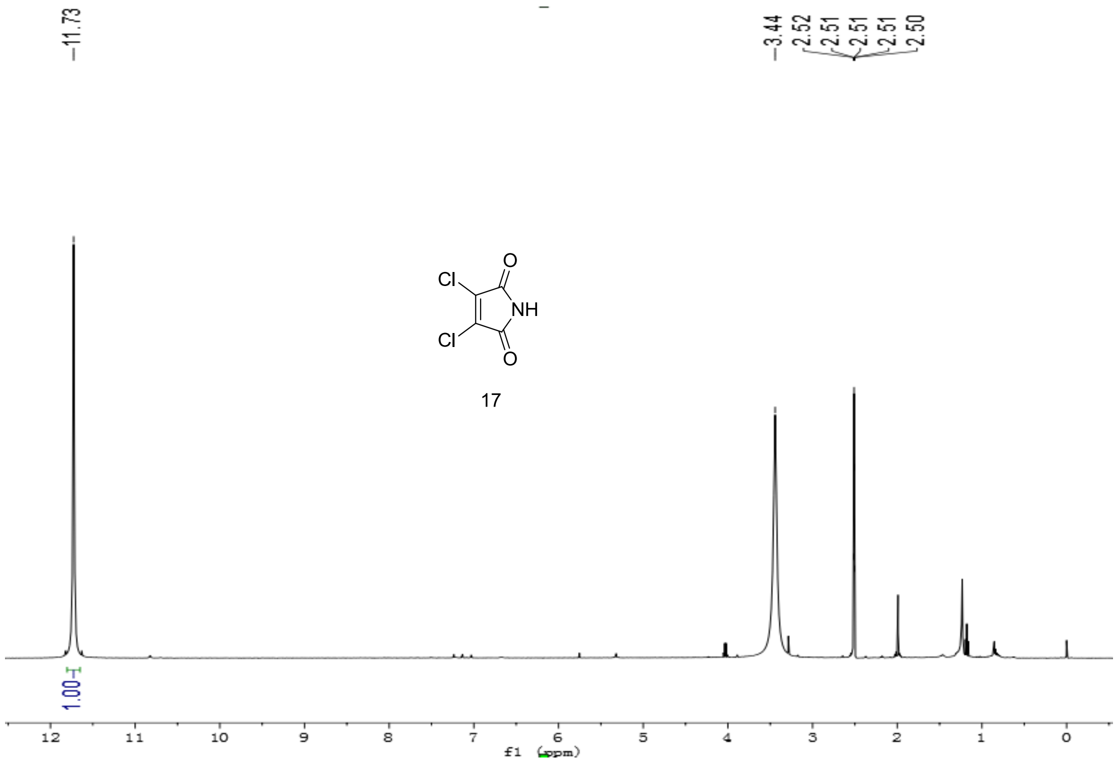

Figure S33 ^{1}H NMR spectra of compound 17

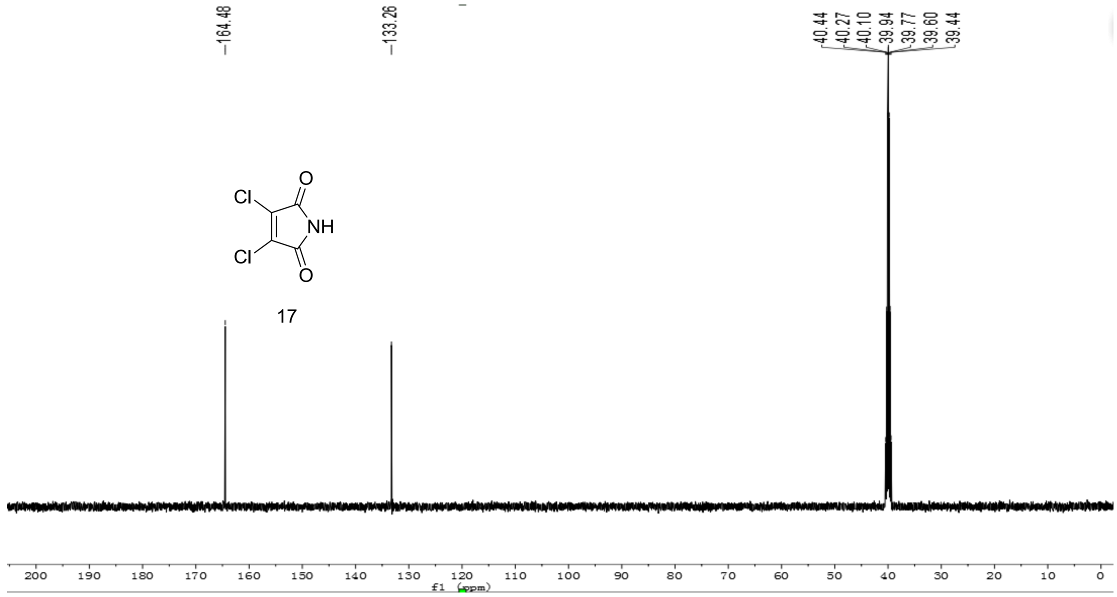

Figure S34 ^{13}C NMR spectra of compound 17
